# Room-Temperature Intermolecular Hydroamination of Vinylarenes Catalyzed by Alkali-Metal Ferrate Complexes

**DOI:** 10.1021/acsorginorgau.4c00066

**Published:** 2024-11-11

**Authors:** Andreu Tortajada, Eva Hevia

**Affiliations:** Departement für Chemie, Biochemie und Pharmazie, Universität Bern, 3012 Bern, Switzerland

**Keywords:** hydroamination, iron, sodium, ferrate, catalysis, bimetallic

## Abstract

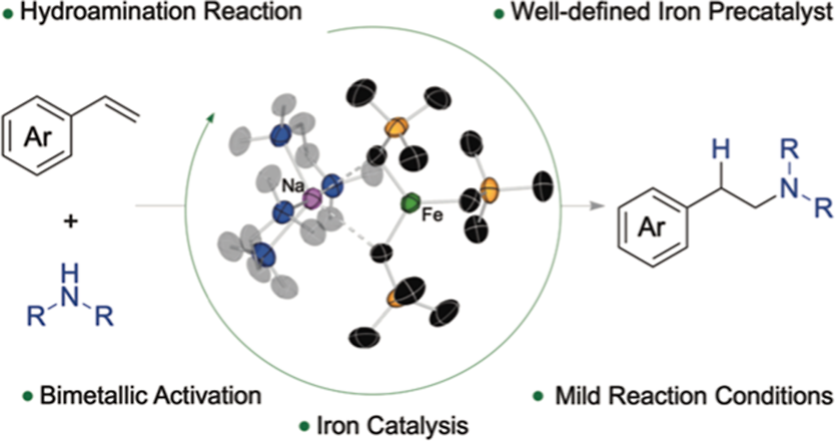

Alkene hydroamination of multiple bonds represents a valuable and atom-economical approach to accessing amines, using simple and widely available starting materials. This reaction requires a metal catalyst, and despite the success of noble transition metals, s-block, or f-block elements, iron organometallic complexes have found limited applications. Partnering iron with an alkali metal and switching on bimetallic cooperativity, we report the synthesis and characterization of a series of highly reactive alkali-metal alkyl ferrate complexes, which can deprotonate amines and activate them toward the catalytic hydroamination of vinylarenes. An alkali-metal effect has been observed, with the sodium analogue being the best for an efficient hydroamination of different styrene derivatives and amines. Stoichiometric studies on the reaction of the sodium tris(alkyl) ferrate complex with 3 mol equiv of piperidine evidenced the ability of the three alkyl groups on Fe to undergo amine metalation, furnishing a novel tris(amido) sodium ferrate which is postulated as a key intermediate in these catalytic transformations. The enhanced reactivity of these alkali-metal ferrates contrasts sharply with that of the Fe(II) bis(alkyl) precursor which is completely inert toward alkene hydroamination.

## Introduction

**Figure 1:**
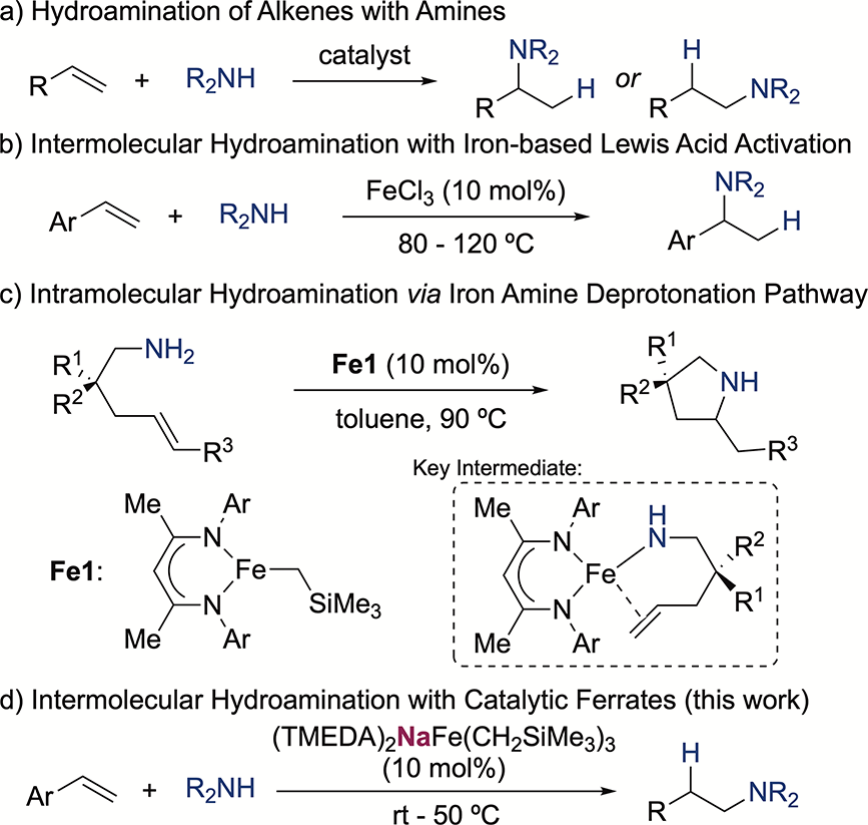
Hydroamination of unsaturated systems, (a) general hydroamination of alkenes, (b) intermolecular hydroamination with Lewis acid catalysis, (c) intramolecular hydroamination, and (d) intermolecular hydroamination with ferrates (this work).

Amines are cornerstone chemical compounds, found in many natural products, agrochemicals, pharmaceuticals, polymers, or dyes.^1,2^ A direct and atom-economical way to prepare these compounds is the hydroamination of multiple carbon–carbon bonds, in which a N–H unit is added into a C–C unsaturated bond, forming a C–H and a C–N bond (Figure 1a). This reaction is very useful to form tertiary amines from readily available starting materials such as alkenes and secondary amines. However, despite being generally thermoneutral or thermodynamically favorable reactions (depending on the substrates used),^3^ they usually require a metal catalyst because they are not kinetically favorable (alkenes and amines are both considered electron-rich).^4^ For said catalytic activation, different strategies have been reported such as the activation of the π-system via coordination (typical for late transition metals),^5,6^ or the activation of the amine moiety via deprotonation to form a nucleophilic metal amide species (typical of s-block metals, lanthanides and actinides).^7^

Despite the advances in this field, the use of iron-based catalysts for activating amine N–H bonds and promoting catalytic hydroamination reaction is still largely underdeveloped.^8−12^ This is somehow surprising considering that Earth-abundant metal catalysis, and in particular using iron compounds, continues to attract widespread interest as a more sustainable alternative to traditional precious metal catalysis, with the added advantages of having lower price, being more environmentally benign and exhibiting generally lower toxicity.^13,14^ Regarding iron catalyzed hydroamination of alkenes, iron(III) chloride has been used as Lewis acid activator, promoting the formation of the aminated Markovnikov product (Figure 1b).^15−17^ Organometallic complexes of iron have also been shown to activate the N–H amine moiety and promote intramolecular hydroamination reactions. Thus, Hannedouche and co-workers have reported the use of β-diketiminate iron(II) alkyl complexes (**Fe1**) for catalytic hydroamination of primary amines tethered to alkenes, leading to the formation of 5- and 6-membered ring heterocycles (Figure 1c).^18−20^ This methodology is characterized by the formation of an iron(II) amidoalkene via deprotonation of the N–H bond, which after alkene migratory insertion into the iron amido bond forms the desired C–N bond. However, this approach was limited to the intramolecular hydroamination of primary amines with geminal disubstitution, narrowing the applicability of these systems to synthesize amines.

Inspired by this work and with the aim of exploring new ways of amine activation with iron-based complexes, we wondered about the possibility of forming more reactive bimetallic alkali metal/iron ate complexes (ferrates). This strategy has been previously exploited by our group in magnesium-catalyzed alkene hydroamination, showing that while neutral Mg dialkyl complexes are completely inert toward this reaction, using potassium magnesiate as a precatalyst allows for the effective hydroamination of styrene type molecules with a range of amines at room temperature in almost quantitative yields.^21^ Within stoichiometric transformations, the alkali-metal magnesiate amido complex could be isolated, showing the deprotonation of the amine motive to form a high order magnesiate, which was proposed as part of the catalytic cycle.

Regarding the use of ferrates in synthesis, they have been proposed and observed as key intermediates in cross-coupling reactions, showing their higher activity to promote these C–C bond formation processes.^22−28^ Their use have not been limited to catalysis, and they have been employed as well in stoichiometric transformations, where our group have also demonstrated their higher reactivity toward deprotonative metalation.^29−33^ For example, we could show that NaFe(HMDS)_3_, HMDS = 1,1,1,3,3,3-hezamethyldisilazide, is able to metalate fragile fluoroarenes in high yields, it being remarkable that the monometallic counterparts Fe(HMDS)_2_ and NaHMDS cannot deliver the metalated intermediates in an efficient manner. However, their low nucleophilicity made us consider a different approach to access bimetallic iron complexes with less bulky and more nucleophilic amines such a piperidine. We envisioned that alkali metal trialkyl ferrate complexes would be basic enough to form in situ the corresponding alkali metal amide ferrates, which in turn could be able to catalyze the hydroamination of styrene type molecules and give the anti-Markovnikov aminated product (Figure 1d). Furthermore, the presence of an alkali-metal can also contribute to the activation of the olefin acting as a built-in Lewis acid,^34,35^ while the iron center would increase the stability of the reaction intermediates, forming less polar Fe–N (or Fe–C) bonds, avoiding side reactions such as polymerization.

## Results and Discussion

**Figure 2:**
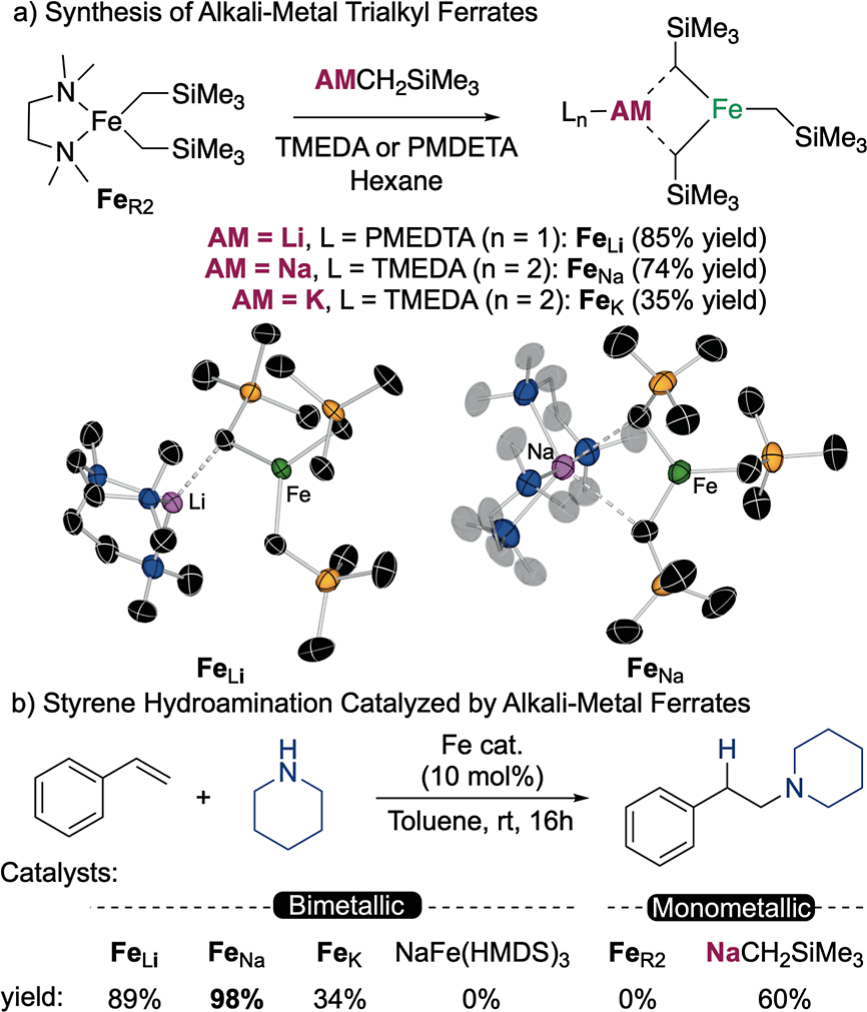
Bimetallic iron complexes in alkene hydroamination. (a) Synthesis of alkali metal trialkyl ferrates, ellipsoids are displayed at 50% probability, and all H atoms have been omitted for clarity. (b) Styrene hydroamination catalyzed by iron organometallic complexes. Conditions: styrene (0.2 mmol), piperidine (0.25 mmol), catalyst (0.02 mmol), and toluene (0.5 mL). Yields were measured by ^1^H NMR spectroscopy using hexamethylbenzene as the internal standard.

For the synthesis of bimetallic trialkyl iron(II) complexes, the –CH_2_SiMe_3_ group was chosen due to its higher steric bulk and the absence of β-hydrogens, limiting the possible decomposition pathways. A few examples of alkyl and aryl iron(II) and (III) ate complexes have been isolated and characterized recently, mostly in the context of cross-coupling reactions with organolithium or Grignard reagents, being prepared from adding an excess of organometallic reagent into an iron salt precursor.^22−28^ In our case, we opted instead for a cocomplexation approach, since we predicted that the organometallic compounds would have better solubility and stability in apolar hydrocarbon solvents. Starting from (TMEDA)Fe(CH_2_SiMe_3_)_2_^36,37^ we added an equivalent of the corresponding AMCH_2_SiMe_3_ (AM = Li, Na, K) and the Lewis donor TMEDA (*N*,*N*,*N*′,*N*′-tetramethylethylenediamine) or PMDETA (*N*,*N*,*N*′,*N*″,*N*″-pentamethyldiethylenetriamine) in hexane to obtain the corresponding bimetallic iron–ate complexes in moderate to good yields (Figures 2a and S9). In the case of the lithium analogue, the tridentate donor PMDETA was optimal to complete the coordination sphere, whereas with the bigger sodium and potassium analogues, two equiv of TMEDA completed the coordination of the alkali metal cations. The corresponding iron complexes **Fe**_**Li**_, **Fe**_**Na**_, and **Fe**_**K**_ could be isolated as off-white crystalline solids in 85%, 74%, and 35% yield, respectively. We observed partial decomposition of the potassium analogue during their synthesis due to the high reactivity of the heavier alkali metal alkyl reagents, explaining the lower yield obtained. Saturated solutions of the compounds in hexane were stored at −30 °C for 16 h to give single crystals suitable for X-ray crystallography, showing very similar trialkyl iron(II) units with Fe–C average distances of 2.087, 2.082, and 2.083 Å for **Fe**_**Li**_, **Fe**_**Na**_, and **Fe**_**K**_, respectively. The alkali metal in these complexes shows contact with the carbon atom(s) of the trialkyl iron unit, where the lithium analogue forms a contacted ion-pair with one of the –CH_2_SiMe_3_ groups with a Li–C distance of 2.400 Å, whereas the sodium and potassium ferrates form a ring-closed contacted ion pair motif, with contact with two –CH_2_SiMe_3_ groups in an average AM–C distance of 3.102 Å (AM = Na) and 3.227 Å (AM = K). Similar contacted ion pair motifs are found in Zn, Mg, or Mn-ate complexes, where (PMDETA)LiM (CH_2_SiMe_3_)_3_ (M = Zn, Mg) shows a single contact with an alkyl group, whereas (TMEDA)_2_AMM(CH_2_SiMe_3_)_3_ (AM = Na, K; M = Zn, Mn) show two contacts with different alkyl groups.^38−40^ Solution NMR characterization in deuterated benzene showed, in every case, very similar chemical shifts for the -SiMe_3_ groups in the ^1^H NMR spectra around 5 ppm (see Supporting Information for details). The determination of the magnetic moment with the Evans method suggests that they are all high spin Fe(II) centers, with solution effective magnetic moments of 5.46, 5.71, and 5.38 μB for **Fe**_**Li**_, **Fe**_**Na**_, and **Fe**_**K**_, respectively, similar to the magnetic moment of 5.7 μB reported for [Fe(II)Bn_3_]^−^.^23^

With these complexes in hand, we proceeded to test their catalytic activity in hydroamination reactions choosing styrene and piperidine as model substrates due to their known reactivity using alkali metals, alkaline-earth metals, or lanthanide-based catalysts.^41−47^ Gratifyingly, the iron ferrates showed excellent catalytic activities when used in 10 mol % loading and using toluene as a solvent at room temperature. Revealing a marked alkali-metal effect, **Fe**_**Li**_ and **Fe**_**Na**_ yielded the best results, with the latter being slightly more active and reaching full conversion after 16 h. **Fe**_**K**_ delivered lower yields of the hydroaminated product with relatively high conversions of styrene, hinting that other decomposition pathways were operating. For comparison, the bimetallic NaFe(HMDS)_3_ was also tested, but it showed no catalytic activity, presumably due to the higher steric demand of the HMDS units and an incomplete transamidation.^48^ The monometallic precursors were also tested in this conditions, showing that (TMEDA)Fe(CH_2_SiMe_3_)_2_ (**Fe**_**R2**_) was not active for the hydroamination of styrene and that NaCH_2_SiMe_3_ catalyzed this transformation,^49^ but the yield obtained was lower with full conversion of the styrene starting material, suggesting that this more reactive catalyst produced a higher amount of side products under this reaction conditions. Moving to more coordinating solvents such as THF slightly slowed down the reaction, but a yield of 94% was obtained when the reaction was heated to 50 °C for 16 h (see Supporting Information for further details).

**Figure 3:**
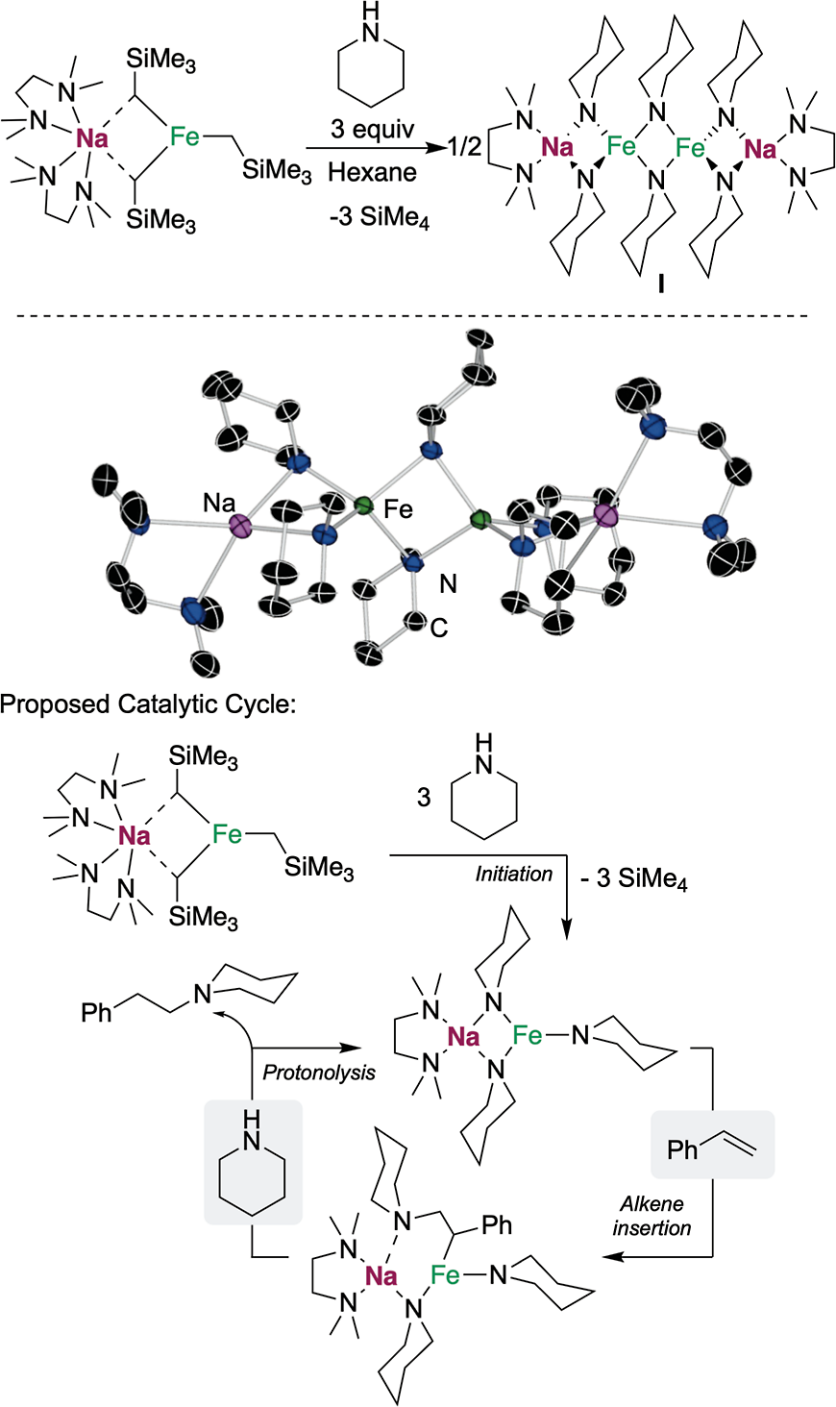
Synthesis and crystal structure of [(TMEDA)NaFe(C_5_H_10_N)_3_]_2_ (**I**) and the proposed catalytic cycle. In the molecular structure, ellipsoids are displayed at 50% probability, and all H atoms have been omitted for clarity.

To try to identify the intermediate species in the hydroamination reaction, we performed stoichiometric experiments of the bimetallic complex **Fe**_**Na**_ with piperidine in a mixture of hexane/toluene as the solvent. It showed a fast reaction that upon cooling to −30 °C delivered deep red crystals. X-ray diffraction analysis of the crystalline solid revealed the formation of a tetranuclear complex [(TMEDA)NaFe(C_5_H_10_N)_3_]_2_ (**I**) formed by two iron and two sodium centers, connected by six piperidide fragments (Figure 3). Adopting a slightly bent Na···Fe···Fe···Na disposition with an average Na···Fe···Fe angle of 164°, each Na atom is chelated by a TMEDA molecule, whereas the Fe centers exhibit distorted tetrahedral geometries coordinating to four piperidide groups. This complex can be envisaged as the piperidide analogue of **Fe**_**Na**_, where now the lower steric demand of the amide substituents favors dimerization to form this tetrametallic complex in the solid state, reminiscent of the magnesium analogue.^21^ However, the two metallic centers in this complexes are relatively closer than in the magnesium case, *d*_Fe–Fe_ = 2.8739(3) Å and *d*_Mg–Mg_ = 2.9764(8) Å, which could indicate that the transition metal character of iron allows some orbital interaction, as similar Fe–Fe distances have been reported for other iron dimers.^50,51^ In solution, an effective magnetic moment of 5.45 μB might suggest that the tetrameric unit breaks to form isolated bimetallic sodium ferrate moieties with a high spin Fe(II) center. When used as catalyst of the hydroamination reaction of styrene with piperidine, we observe a comparable yield of 93% of **1a** after 16 h at room temperature, and therefore, we posit that the formation of this trisamido sodium ferrate in solution occurs rapidly and these species are part of the catalytic cycle. We propose that in solution, the trisamido sodium ferrate is able to insert the vinylarene and form an alkyl iron intermediate, which can be protonated by another molecule of amine to close the catalytic cycle. This was further supported by a crossover experiment, in which styrene was reacted with morpholine using **I** (10 mol %) as catalyst, obtaining 14% of **1a** and 78% of **1g** (see Supporting Information for details), which further supports the involvement of the bimetallic sodium ferrates in the hydroamination reaction and not the monometallic sodium amides. To test the important role of the alkali metal, when 15-crown-5 was added to the catalytic reaction, a reduced yield of 23% of the corresponding hydroaminated product was obtained, which together with the alkali metal effect reported before confirms the importance of the alkali-metal for an efficient hydroamination.

**Figure 4:**
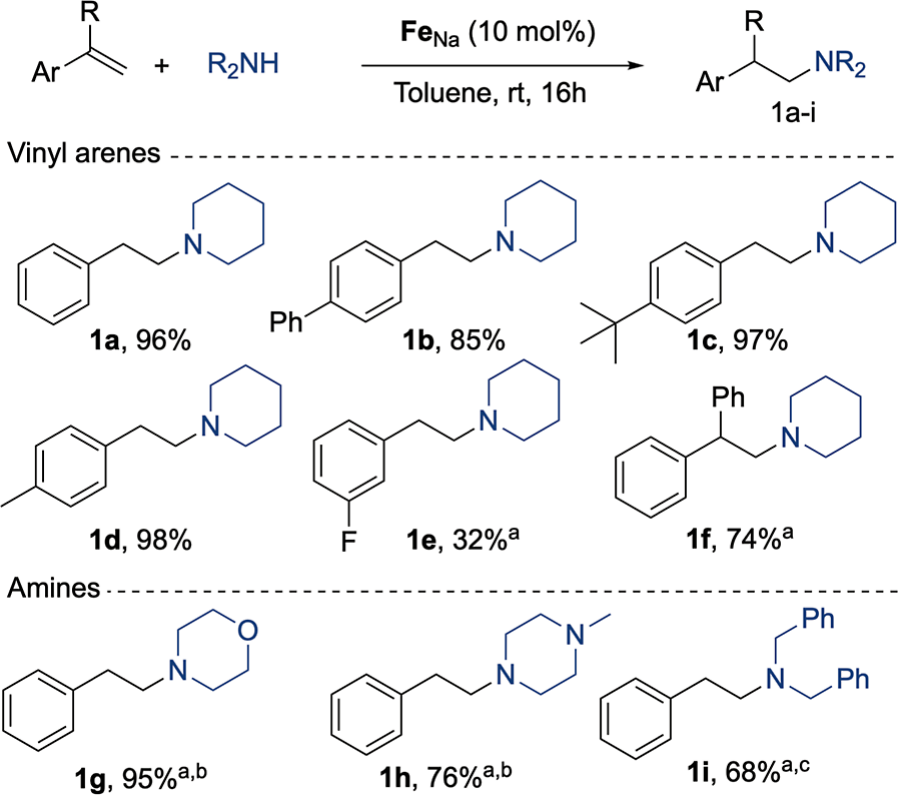
Hydroamination of styrene derivatives with secondary amines. Conditions: vinylarene (0.2 mmol), amine (0.25 mmol), **Fe**_**Na**_ (0.02 mmol), toluene (0.5 mL), rt, 16 h. (a) Reaction performed at 50 °C, (b) reaction performed in THF, and (c) NMR yield using C_6_Me_6_ as the internal standard.

With this information in hand, we tested the substrate scope of the reaction (Figure 4). Different styrene type molecules were used, observing good to excellent yields of hydroaminated product with a phenyl (**1b**), a *tert*-butyl (**1c**), or a methyl substituent (**1d**) in the aromatic ring. Other more sensitive groups such as fluorines were tolerated, albeit the hydroaminated product (**1e**) was obtained with a low yield. Substitution in the double bond with another phenyl group delivered the corresponding hydroaminated product (**1f**) in 74% yield. Moving to other amines, we observed that morpholine and *N*-methylpiperazine could be used instead of piperidine to obtain the hydroaminated products in excellent yields (**1g**, **1h**). However, in this case, the reaction had to be performed using THF as solvent at 50 °C due to the high insolubility of the intermediate species in toluene. Noncyclic amines such as dibenzylamine delivered as well the hydroaminated product (**1i**) in good yields, showing that the system is not restricted to cyclic amines. Less nucleophilic amines such as *N*-methylaniline or diphenylamine failed to deliver the hydroamination of styrene, even when heated to higher reaction temperatures, presumably due to their lower nucleophilicity. From the alkene substrate scope, this protocol also remains limited to activated olefins, observing no hydroaminated product when terminal unactivated olefins (i.e., 1-octene) or alkynes (i.e., diphenylacetylene) were reacted at 80 °C for 16 h.

## Conclusions

Overall, the synthesis and characterization of alkali metal ferrates with alkyl substituents have allowed the development of a new intermolecular hydroamination protocol of styrenes with secondary amines. The more basic alkyl substituents on iron have been able to deprotonate the amine motive, a compound that we have been able to isolate and characterize by X-ray crystallography and NMR spectroscopy when using piperidine. In catalysis, a marked alkali-metal effect can be observed, highlighting the importance of the partnership of both metals. The sodium ferrate showed the best results, whereas the lithium analogue delivered lower rates and the potassium ferrate promoted other unproductive pathways. It is remarkable that the combination of both metals was necessary to obtain high yields of the product, since the dialkyl iron complex did not promote the reaction and the very reactive alkyl sodium formed a higher amount of side products. The hydroamination protocol could be extended to other styrene derivatives and secondary amines, showing the possibilities of this new approach for the synthesis of tertiary amines and broadening the catalogue of iron catalyzed transformations. The partnership of iron with other abundant metals such as alkali metals can open new reactive pathways, showing that this strategy is not limited to the main group elements and can be extended to transition metals.

## Experimental Section

### General

All procedures were conducted using standard Schlenk line and glovebox techniques under an inert atmosphere of argon. Hexane was degassed, purified, and collected via an MBraun SPS 5 and stored over 4 Å molecular sieves for at least 24 h prior to use. THF was dried by heating to reflux over sodium wire/benzophenone ketyl radical and stored over 4 Å molecular sieves for 24 h prior to use. Deuterated solvents (C_6_D_6_ and C_7_D_8_) were purchased from Sigma-Aldrich or Eurisotope, dried over NaK alloy for 16 h, and then cycled through three rounds of degassing by employing a freeze–pump–thaw method. The deuterated solvents were then collected via vacuum transfer and stored under an argon atmosphere over 4 Å molecular sieves. All substrates employed in this study are commercially available and were used as received (solids) or degassed by freeze–pump–thaw and stored over molecular sieves (liquids).

NMR spectra were recorded on Bruker spectrometers operating at 300, 400, or 500 MHz. Elemental analyses (C, H, and N) were conducted with a Flash 2000 organic elemental analyzer (Thermo Scientific). Samples were prepared in the glovebox under an argon atmosphere and sealed in an airtight container prior to analyses. All results were obtained from the Analytical Research and Services Schürch Group of the University of Bern. Samples were weighed on a Mettler Toledo balance with ±2 μg resolution, and sample weights from 1 to 3 mg were used. For calibration, a reference material such as cysteine was used. The presented values are the average of determinations in triplicate to ensure consistency.

### Synthesis of Alkali-Metal Ferrates

#### (PMDETA)LiFe(CH_2_SiMe_3_)_3_ (Fe_Li_)

(TMEDA)Fe(CH_2_SiMe_3_)_2_ (1 mmol, 346.5 mg) and LiCH_2_SiMe_3_ (1 mmol, 94.2 mg) were dissolved in 10 mL of hexane. After a few seconds, it formed a light-yellow oil, to which PMDETA (2 mmol, 0.42 mL) was added, and the solvent reduced to a third of the original volume. The mixture was cooled to −30 °C in a bath to form a light brown solid. The supernatant was removed with a syringe, and the solid was washed twice with 3 mL of cold hexane. The resulting solid was dried under a vacuum to obtain a light brown solid (423.4 mg, 85% yield). ^1^H NMR (300 MHz, C_6_D_6_): δ 5.93–3.48 (m, 27H), −0.30 – −10.19 (m, 23H), ^7^Li NMR (117 MHz, C_6_D_6_): δ 4.48. Elemental analysis: Anal. Calcd for C_21_H_56_FeLiN_3_Si_3_: C, 50.68; H, 11.34; N, 8.44; Found: C, 50.86; H, 11.65; N, 8.86.

#### (TMEDA)_2_NaFe(CH_2_SiMe_3_)_3_ (Fe_Na_)

(TMEDA)Fe(CH_2_SiMe_3_)_2_ (2 mmol, 693 mg), NaCH_2_SiMe_3_ (2 mmol, 220.4 mg), and TMEDA (2 mmol, 0.300 mL) were dissolved in 15 mL of hexane at 0 °C. The mixture was stirred for 30 min, and a colorless precipitate formed. The supernatant was removed with a canula filtration, the solid was further washed with cold hexane, and the resulting solid was dried under vacuum. The desired compound was obtained as an off-white solid (851 mg, 74% yield). ^1^H NMR (300 MHz, C_6_D_6_): δ 4.88 (s, 27H), 1.56 – −2.31 (m, 32H). Elemental analysis: Anal. Calcd for C_24_H_63_FeN_4_NaSi_3_: C, 50.32; H, 11.44; N, 9.78. Found: C, 50.18; H, 11.43; N, 9.93.

#### (TMEDA)_2_KFe(CH_2_SiMe_3_)_3_ (Fe_K_)

(TMEDA)Fe(CH_2_SiMe_3_)_2_ (1 mmol, 346.5 mg), KCH_2_SiMe_3_ (1 mmol, 126.3 mg), and TMEDA (1 mmol, 0.150 mL) were dissolved in 20 mL of hexane at 0 °C. A black solid was formed, and the supernatant was filtered via canula to remove this solid. After removing around half of the solvent under vacuum, a solid started to form. Further cooling down the mixture to 0 °C delivered a colorless solid. The supernatant was removed via canula filtration, and the solid was washed with cold hexane to deliver a crystalline light brown solid (203 mg, 35% yield). ^1^H NMR (300 MHz, C_6_D_6_): δ 6.36–3.84 (m, 27H), 1.08 – −1.44 (m, 32H). Elemental analysis: Anal. Calcd for C_24_H_63_FeN_4_KSi_3_: C, 48.94; H, 11.12; N, 9.51. Found: C, 48.74; H, 11.18; N, 9.54.

#### [(TMEDA)NaFe(C_5_H_10_N)_3_]_2_ (**I**)

The complex (TMEDA)_2_·NaFe(CH_2_SiMe_3_)_3_ (0.2 mmol, 114 mg) was dissolved in 3 mL of toluene in the glovebox. To this solution, piperidine (0.6 mmol, 60 μL) was added dropwise to form a brown/red solution. Addition of 2 mL of hexane and cooling down to −30 °C for 16 h delivered dark red crystals, that were suitable for X-ray crystallography. Removal of the supernatant and washing of the solid with cold pentane rendered a brown solid as the desired product (55.5 mg, 62% yield). ^1^H NMR (300 MHz, C_6_D_6_): δ 31.45–22.18 (m), 16.06–12.10 (m), 1.72–1.07 (m), 1.07–0.71 (m), 0.47 – −0.54 (m). Elemental analysis: Anal. Calcd for C_42_H_92_Fe_2_N_10_Na_2_: C, 56.37; H, 10.36; N, 15.65. Found: C, 55.92; H, 10.48; N, 15.32.

### Catalytic Hydroamination with Alkali-Metal Ferrates

To a J-young NMR tube or a vial was added the catalyst (0.02 mmol, 10 mol %) in a glovebox, and it was dissolved in toluene (or THF). The amine (0.25 mmol) and the vinylarene (0.2 mmol) were then added, and the reaction was left at room temperature for 16 h. After that time, the reaction vessel was opened to air, diluted with EtOAc, and filtered through silica gel. The organic solvent was removed under reduced pressure to give the crude compounds, which, after purification by column chromatography, delivered the desired hydroaminated products.

## Data Availability

The data underlying this study are available in the published article and its Supporting Information.

## Supporting Information Available

Experimental details, NMR spectra, and X-ray crystallog-raphy data General methods, synthesis of organometallic complexes, X-ray crystallographic data, stoichiometric reaction with NaFe(HMDS)_3_, catalytic reactions, cross-over experiment with I and morpholine, characterization of the products, and copies of NMR Spectra (PDF)
