# Supplementary material for: Room-Temperature Intermolecular Hydroamination of Vinylarenes Catalyzed by Alkali-Metal Ferrate Complexes
Source: ACS Org Inorg Au. 2024 Nov 11;5(1):62–8. doi: 10.1021/acsorginorgau.4c00066 (PMC11803465; doi:10.1021/acsorginorgau.4c00066)
Supplement: Supplementary file 1 — gg4c00066_si_001.pdf [file gg4c00066_si_001.pdf]

Room Temperature Intermolecular Hydroamination of Vinylarenes Catalyzed by Alkali-Metal Ferrate Complexes

Andreu Tortajada, Eva Hevia*

Departement für Chemie, Biochemie und Pharmazie, Universität Bern, 3012 Bern, Switzerland

Table of Contents

General Methods *3*

Synthesis of Organometallic Complexes *4*

Synthesis of $NaCH_2SiMe_3$ 4

Synthesis of KCH_2SiMe_3 4

Synthesis of (TMEDA)Fe(CH_2SiMe_3)$_2$ 4

Synthesis of (PMDETA)LiFe(CH_2SiMe_3)$_3$ (Fe_{Li}) 5

Synthesis of (TMEDA)$_2$NaFe(CH_2SiMe_3)$_3$ (Fe_{Na}) 7

Synthesis of (TMEDA)$_2$KFe(CH_2SiMe_3)$_3$ (Fe_K) 8

Synthesis of [(TMEDA)NaFe($C_5H_{10}N$)$_3$]$_2$ (I) 9

X-Ray Crystallographic Data *10*

Stoichiometric Reaction with NaFe(HMDS)$_3$ *15*

Catalytic Reactions *16*

Typical Procedure 16

Cross-over Experiment with I and morpholine 20

Characterization of the products 21

Copies of NMR Spectra 24

References *34*

General Methods

All procedures were conducted using standard Schlenk line and glove box techniques under an inert atmosphere of argon. Hexane was degassed, purified and collected via an MBraun SPS 5 and stored over 4 Å molecular sieves for at least 24 hours prior to use. THF was dried by heating to reflux over sodium-wire/benzophenone ketyl radical and stored over 4 Å molecular sieves for 24 hours prior to use. Deuterated solvents (C_6D_6 and C_7D_8) were purchased from Sigma Aldrich or Eurisotope, dried over NaK alloy for 16 hours and then cycled through three rounds of degassing by employing a freeze-pump-thaw method. The deuterated solvents were then collected via vacuum transfer and stored under argon atmosphere over 4 Å molecular sieves.

All substrates employed in this study are commercially available and were used as received (solids) or degassed by freeze-pump-thaw and stored over molecular sieves (liquids).

The polydentate amines used in this study were dried over calcium hydride, distilled under reduced pressure and stored over molecular sieves prior their use.

NMR spectra were recorded on Bruker spectrometers operating at 300 MHz, 400 MHz or 500 MHz. Spectra were analyzed using MestReNova software and referenced internally to the corresponding residual protium solvent peaks.

Elemental analyses (C, H and N) were conducted with a Flash 2000 Organic Elemental Analyser (Thermo Scientific). Samples were prepared in the glovebox under argon atmosphere and sealed in an air-tight container prior to analyses. All results were obtained by the Analytical Research and Services Schürch Group of the University of Bern. Samples were weighed on a Mettler Toledo balance with ± 2 μg resolution and sample weights from 1-3 mg were used. For calibration, a reference material such as cysteine was used. The presented values are the average of determinations in triplicate to ensure consistency.

Synthesis of Organometallic Complexes

Synthesis of $NaCH_2SiMe_3$

In an argon-filled Schlenk flask, 50.0 mL of dry hexane was added to 2.88 g (30 mmol) of Na*Ot*Bu and 2.82 g (30 mmol) of $LiCH_2SiMe_3$ affording a fine, white suspension which was the cooled to 0 °C and stirred for 1 h. The suspension was warmed to ambient temperature and stirred overnight. Isolation of the white precipitate ($NaCH_2SiMe_3$) was achieved by gravity filtration using a glass-tapped filter frit. The filter cake was washed with 3 x 15 mL aliquots of fresh hexane until the liquors ran completely clear. The solid was dried under vacuum and stored in the glovebox for further use. Typical yield = 89 %, 2.94 g.
For a more detailed procedure, illustrated with pictures of the set-up check our previous publication.[1]

Synthesis of KCH_2SiMe_3

Synthesis of KCH_2SiMe_3 was performed in an analogous way to $NaCH_2SiMe_3$ (see above), using $LiCH_2SiMe_3$ and KOtBu.

Synthesis of (TMEDA)$Fe(CH_2SiMe_3)_2$

Procedure adapted from the literature,[2] using commercially available $LiCH_2SiMe_3$ instead of $ClMgCH_2SiMe_3$:

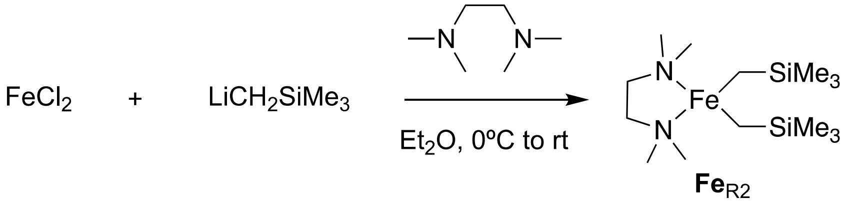

Iron chloride anhydrous (10 mmol, 1.268 g) was suspended in Et_2O under an atmosphere of argon, to which TMEDA (10 mmol, 1.50 mL) was added and it was stirred overnight at room temperature. Next day, the solution was cold to 0 ºC using an ice bath and $LiCH_2SiMe_3$ (20 mmol, 1.883 g) dissolved in Et_2O were added dropwise using a canula. The mixture was stirred for 1 hour and warmed up to room temperature. The solvent was removed under vacuum, condensing the Et_2O in an external trap cooled with liquid nitrogen. The residue was suspended in hexane and stirred for 10 minutes. The solution was allowed to settle, and it was filtered with a canula equipped with a glass filter pad and transferred into a clean Schlenk flask. The solvent was then removed under vacuum, to give a light brown crystalline solid, which was entered into a glovebox and stored at -30 ºC (3.08g, 89% yield). Spectroscopic data agree with the reported in the literature.[2]

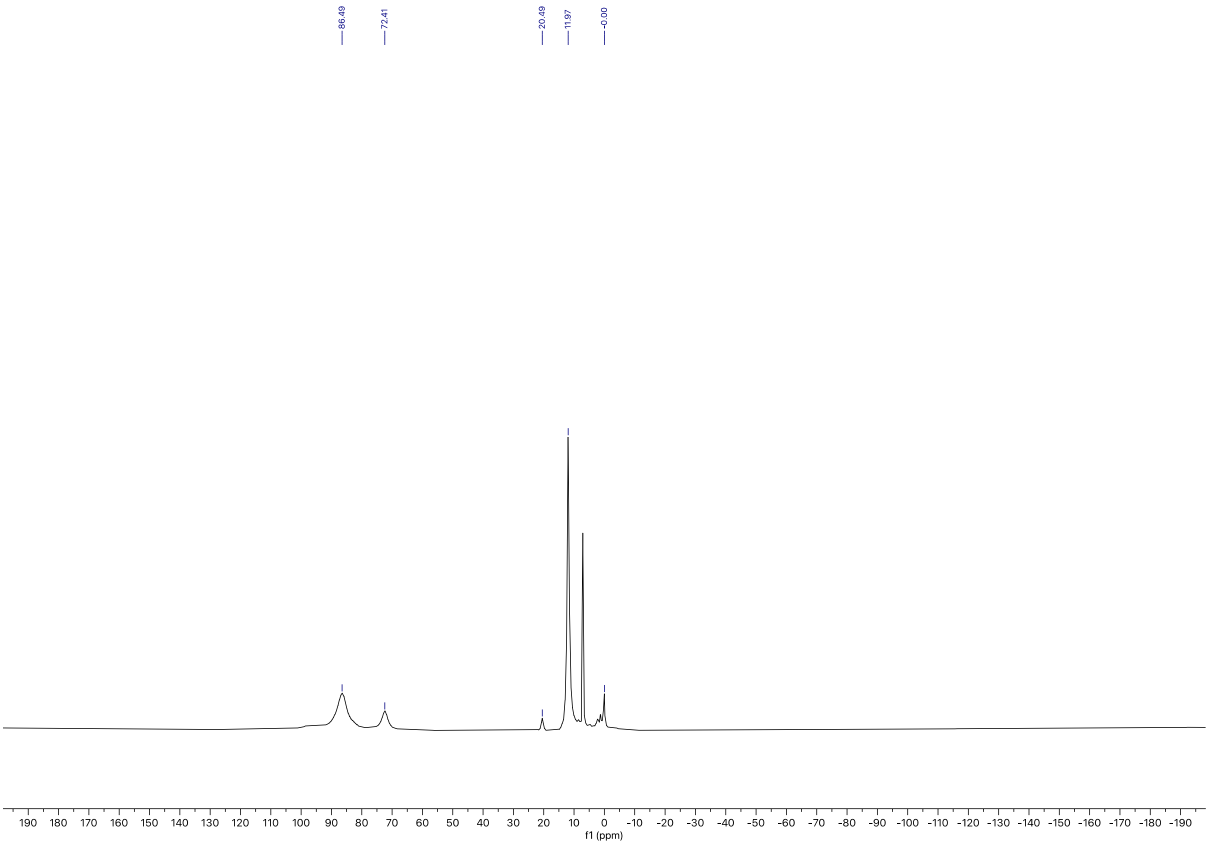

Figure S 1. ^1H NMR spectrum of TMEDA·Fe($CH_2SiMe_3)_2$ in C_6D_6.

Synthesis of (PMDETA)LiFe($CH_2SiMe_3)_3$ (Fe_{Li})

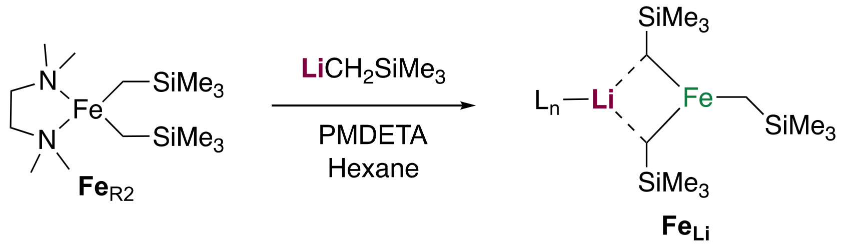

(TMEDA)Fe($CH_2SiMe_3)_2$ (1 mmol, 346.5 mg) and $LiCH_2SiMe_3$ (1 mmol, 94.2 mg) were dissolved in 10 mL of hexane. After a few seconds they formed a light-yellow oil. PMDETA (2 mmol, 0.42 mL) was added to the mixture and the solvent reduced to a third of the original volume. The mixture was cooled down to -30 ºC in a bath to form a light brown solid. The supernatant was removed with a syringe and the solid was washed twice with 3 mL of cold hexane. It was dried under vacuum to obtain a light brown solid (423.4 mg, 85% yield), which was entered into the glovebox. Single crystals were grown from slow diffusion of hexane into a solution of the compound in toluene, which gave colorless crystals.

^{1}H NMR (300 MHz, C_6D_6) δ 5.93 – 3.48 (m, 27H), -0.30 – -10.19 (m, 23H)

^{7}Li NMR (117 MHz, C_6D_6) δ 4.48.

Elemental analysis: Anal. Calcd for $C_{21}H_{56}FeLiN_3Si_3$: C, 50.68; H, 11.34; N, 8.44. Found: C, 50.86; H, 11.65; N, 8.86

Magnetic moment: the solution magnetic moment was measured using the Evans' method with a capilar of benzene, giving a value of 5.46 μB.

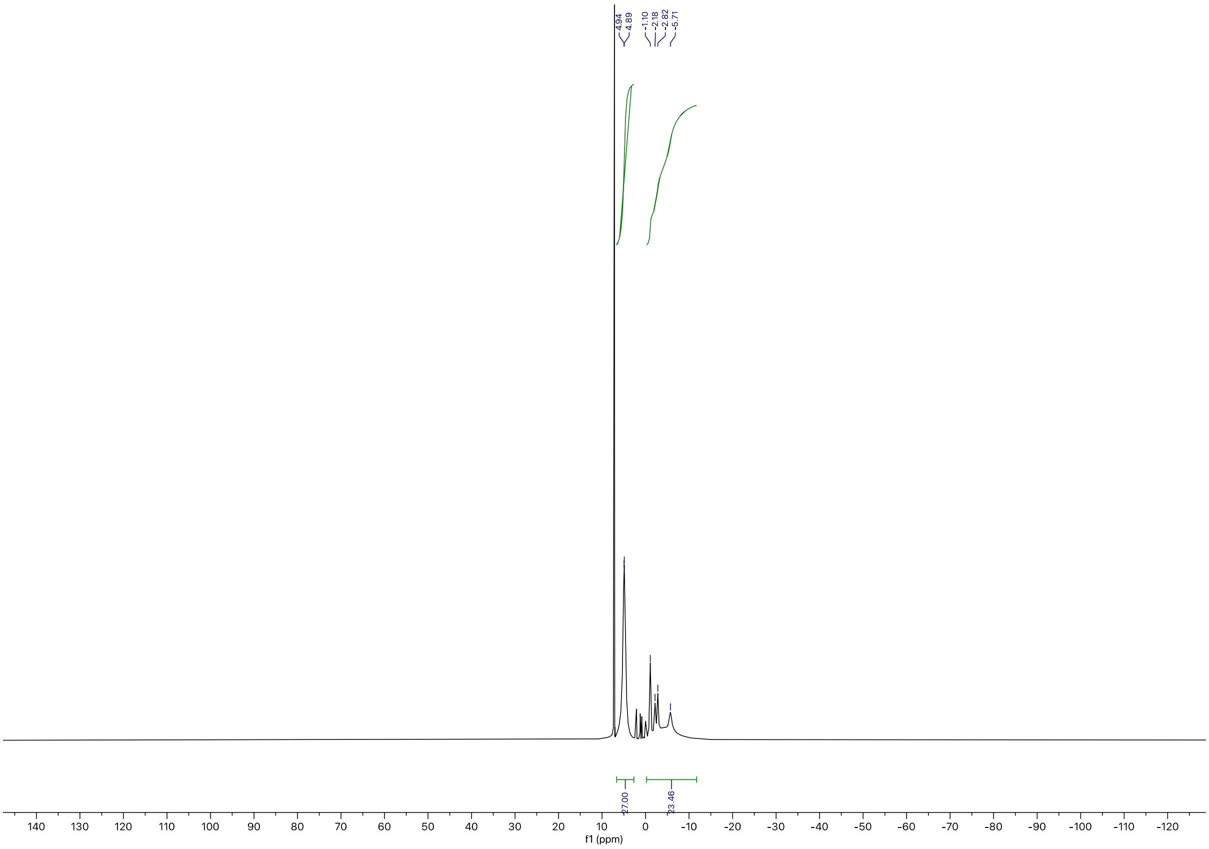

Figure S 2. ¹H NMR spectrum of PMDETA·LiFe(CH$_2$SiMe$_3$)$_3$ in C$_6$D$_6$.

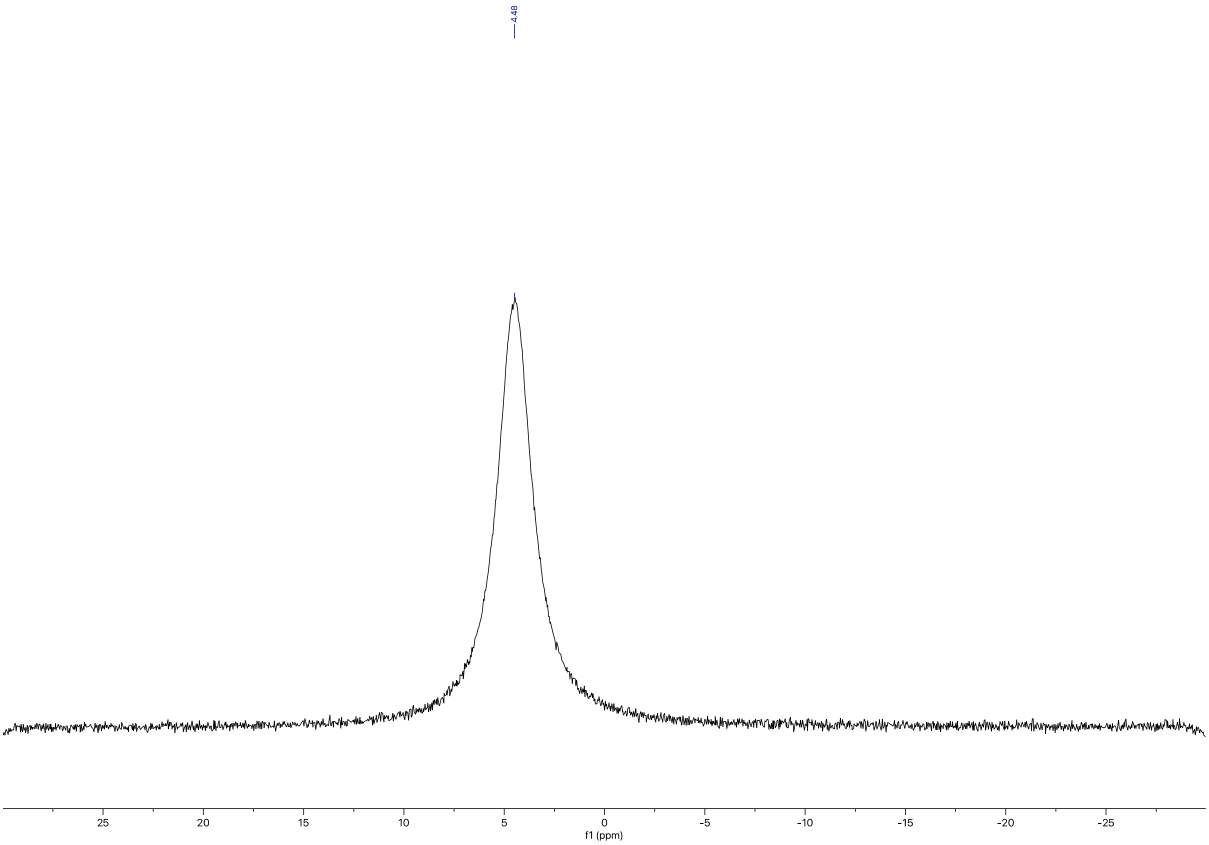

Figure S 3. ⁷Li NMR spectrum of PMDETA·LiFe(CH$_2$SiMe$_3$)$_3$ in C$_6$D$_6$.

Synthesis of $(TMEDA)_2NaFe(CH_2SiMe_3)_3$ (Fe_{Na})

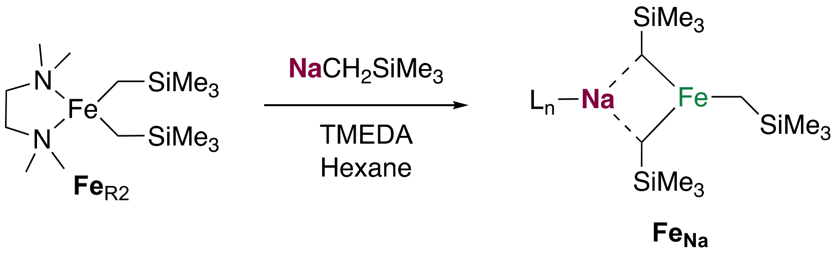

$(TMEDA)Fe(CH_2SiMe_3)_2$ (2 mmol, 693 mg), $NaCH_2SiMe_3$ (2 mmol, 220.4 mg) and TMEDA (2 mmol, 0.300 mL) were dissolved in 15 mL of hexane at 0 ºC. The mixture was stirred for 30 min and a colorless precipitate formed. The supernatant was removed with a canula filtration, the solid was further washed with cold hexane and the resulting solid was dried under vacuum. An off-white solid was obtained as the desired compound (851 mg, 74% yield). A saturated solution in hexanes was cooled down to -30 ºC in the glovebox freezer to give crystals suitable for X-Ray crystallography.

^{1}H NMR (300 MHz, C_6D_6) δ 4.88 (s, 27H), 1.56 – -2.31 (m, 32H).

Elemental analysis: Anal. Calcd for $C_{24}H_{63}FeN_4NaSi_3$: C, 50.32; H, 11.44; N, 9.78. Found: C, 50.18; H, 11.43; N, 9.93.

Magnetic moment: the solution magnetic moment was measured using the Evans' method with a capilar of benzene, giving a value of 5.71 μB.

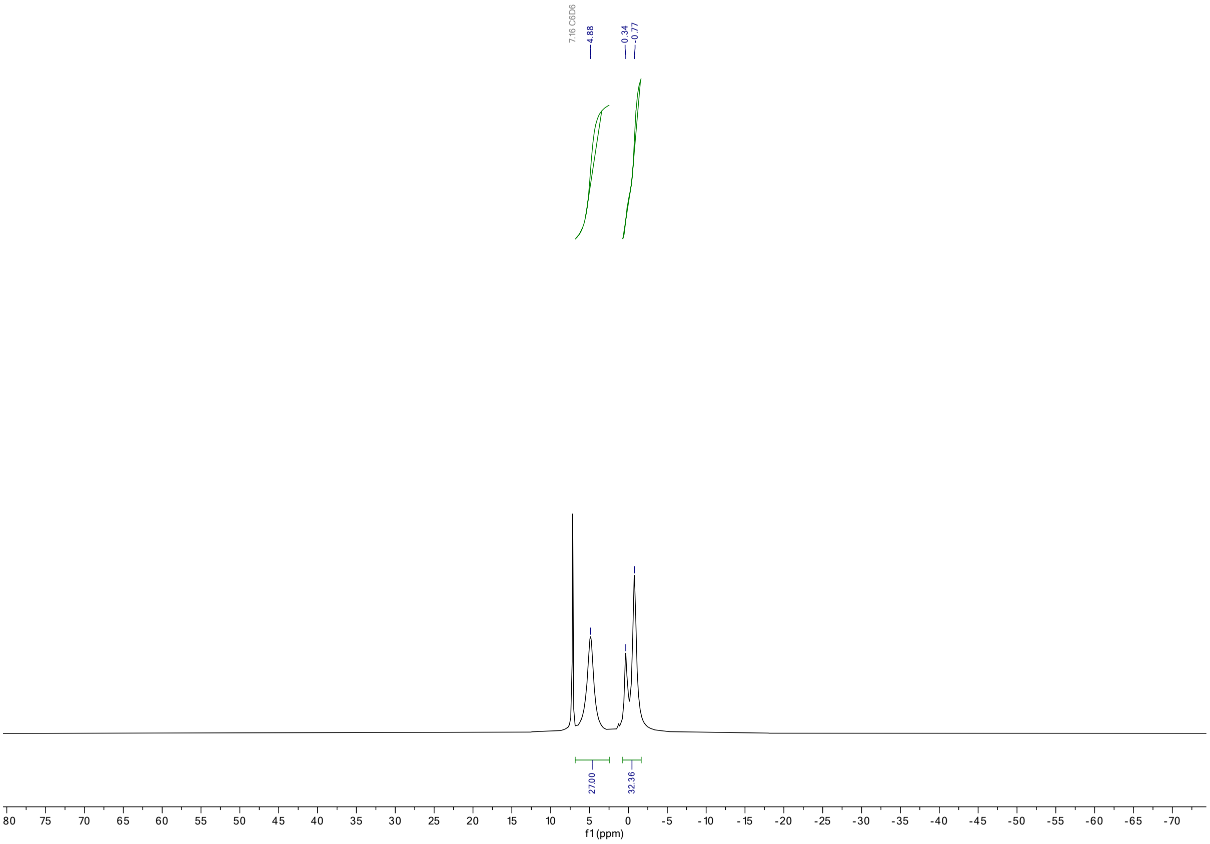

Figure S 4. ^{1}H NMR spectrum of $TMEDA_2{\cdot}NaFe(CH_2SiMe_3)_3$ in C_6D_6.

Synthesis of $(TMEDA)_2KFe(CH_2SiMe_3)_3$ (Fe_K)

$(TMEDA)Fe(CH_2SiMe_3)_2$ (1 mmol, 346.5 mg), KCH_2SiMe_3 (1 mmol, 126.3 mg) and TMEDA (1 mmol, 0.150 mL) were dissolved in 20 mL of hexane at 0 ºC. A black solid was formed, and the supernatant was filtered via canula to remove this solid. After removing around half of the solvent under vacuum, a solid started to form. Further cooling down the mixture to 0 ºC formed a colorless solid. Supernatant was removed via canula filtration, the solid was washed with cold hexane to deliver a crystalline light brown solid (203 mg, 35% yield).

^{1}H NMR (300 MHz, C_6D_6) δ 6.36 – 3.84 (m, 27H), 1.08 – -1.44 (m, 32H).

Elemental analysis: Anal. Calcd for $C_{24}H_{63}FeN_4KSi_3$: C, 48.94; H, 11.12; N, 9.51. Found: C, 48.74; H, 11.18; N, 9.54.

Magnetic moment: the solution magnetic moment was measured using the Evans' method with a capilar of benzene, giving a value of 5.38 μB.

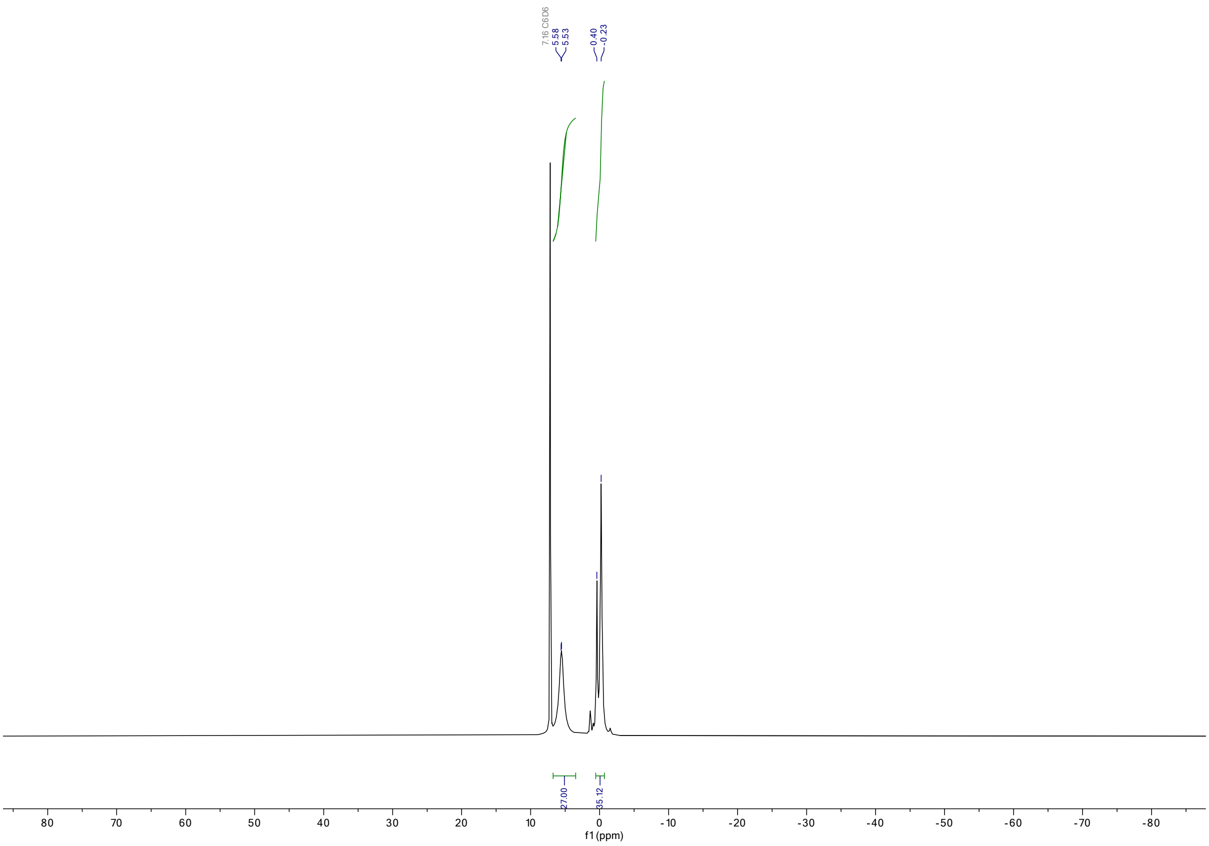

Figure S 5. ^{1}H NMR spectrum of $TMEDA_2 \cdot KFe(CH_2SiMe_3)_3$ in C_6D_6.

Synthesis of $[(TMEDA)NaFe(C_5H_{10}N)_3]_2$ (I)

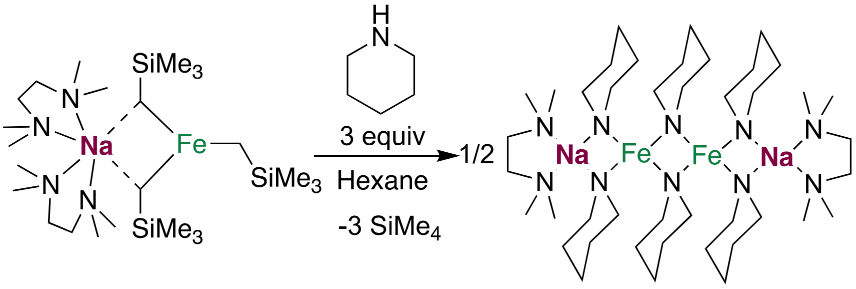

The complex $(TMEDA)_2NaFe(CH_2SiMe_3)_3$ (0.2 mmol, 114 mg) was dissolved in 3 mL of toluene in the glovebox. To this solution, piperidine (0.6 mmol, 60 μL) was added dropwise to form a brown/red solution. Addition of 2 mL of hexane and cooling down to -30 ºC for 16 hours delivered dark red crystals, that were suitable for X-Ray crystallography. Removal of the supernatant and washing of the solid with cold pentane rendered a brown solid as the desired product (55.5 mg, 62% yield).

^{1}H NMR (300 MHz, C_6D_6) δ 31.45 – 22.18 (m), 16.06 – 12.10 (m), 1.72 – 1.07 (m), 1.07 – 0.71 (m), 0.47 – -0.54 (m).

Elemental analysis: Anal. Calcd for $C_{42}H_{92}Fe_2N_{10}Na_2$: C, 56.37; H, 10.36; N, 15.65. Found: C, 55.92; H, 10.48; N, 15.32.

Magnetic moment: the solution magnetic moment was measured using the Evans' method with a capilar of benzene, giving a value of 5.45 μB.

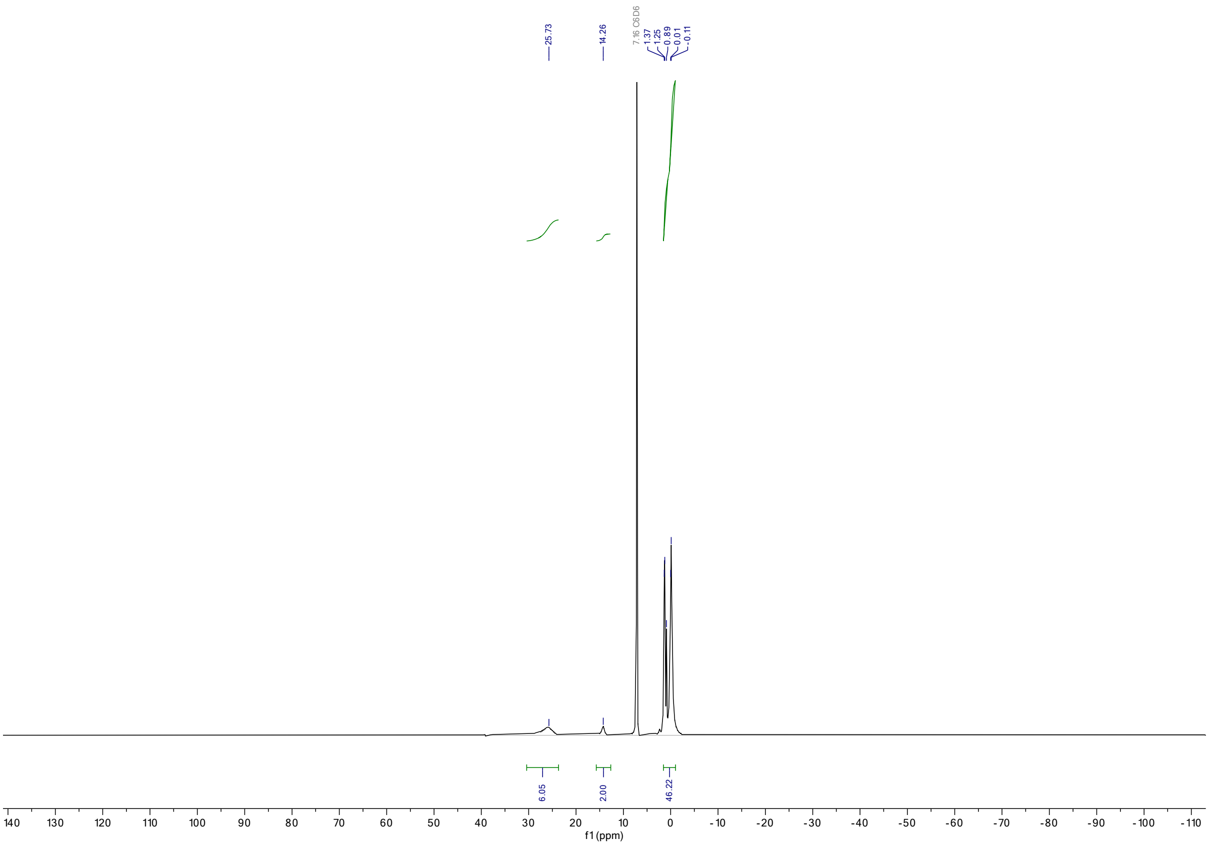

Figure S 6. ^{1}H NMR spectrum of $[(TMEDA)NaFe(C_5H_{10}N)_3]_2$ in C_6D_6.

X-Ray Crystallographic Data

For all the structures:

-Data reduction was performed using the *CrysAlisPro*[5] program. The intensities were corrected for Lorentz and polarization effects, and an absorption correction based on the multi-scan method using SCALE3 ABSPACK in *CrysAlisPro*[5] was applied. Data collection and refinement parameters are given in *Table 1.*

-The structure was solved by intrinsic phasing using *SHELXT*[6], which revealed the positions of all non-hydrogen atoms of the title compound. All non-hydrogen atoms were refined anisotropically. H-atoms were assigned in geometrically calculated positions and refined using a riding model where each H-atom was assigned a fixed isotropic displacement parameter with a value equal to 1.2Ueq of its parent atom (1.5Ueq for methyl groups).

-Refinement of the structure was carried out on F^2 using full-matrix least-squares procedures, which minimized the function $\Sigma w(F_o^2 - F_c^2)^2$. The weighting scheme was based on counting statistics and included a factor to downweight the intense reflections. All calculations were performed using the *SHELXL-2014/7*[7] program in OLEX2.[8]

PMDETA·LiFe(CH_2SiMe_3)$_3$ (Fe_{Li}):

Crystal-Structure Determination. A crystal of $C_{21}H_{56}FeLiN_3Si_3$ immersed in parabar oil was mounted at ambient conditions and transferred into the stream of nitrogen (173 K). All measurements were made on a *RIGAKU Synergy S* area-detector diffractometer[5] using mirror optics monochromated Cu *K*α radiation (λ = 1.54184 Å). The unit cell constants and an orientation matrix for data collection were obtained from a least-squares refinement of the setting angles of reflections in the range 2.383° < θ < 79.859°. A total of 6288 frames were collected using ω scans, with 0.4 second exposure time (1.5 s for high-angle reflections), a rotation angle of 0.5° per frame, a crystal-detector distance of 34.0 mm, at T = 173(2) K.

(TMEDA)$_2$·NaFe(CH_2SiMe_3)$_3$ (Fe_{Na}):

Crystal-Structure Determination. A crystal of $C_{24}H_{65}FeN_4NaSi_3$ immersed in parabar oil was mounted at ambient conditions and transferred into the stream of nitrogen (173 K). All measurements were made on a *RIGAKU Synergy S* area-detector diffractometer[5] using mirror optics monochromated Cu *K*α radiation (λ = 1.54184 Å). The unit cell constants and an orientation matrix for data collection were obtained from a least-squares refinement of the setting angles of reflections in the range 2.593° < θ < 78.414°. A total of 5974 frames were collected using ω scans, with 2.5 second exposure time (20 s for high-angle reflections), a rotation angle of 0.5° per frame, a crystal-detector distance of 34.0 mm, at T = 173(2) K.

Refinement: Disorder model was included for parts of the structure where the occupancies of each disorder component were refined through the use of a free variable. The sum of equivalent components was constrained to 1 i.e. 100%.

(TMEDA)$_2$·KFe(CH$_2$SiMe$_3$)$_3$ (Fe$_K$):

Crystal-Structure Determination. A crystal of $C_{24}H_{65}FeKN_4Si_3$ immersed in parabar oil was mounted at ambient conditions and transferred into the stream of nitrogen (173 K). All measurements were made on a *RIGAKU Synergy S* area-detector diffractometer[5] using mirror optics monochromated Cu *K*α radiation (λ = 1.54184 Å). The unit cell constants and an orientation matrix for data collection were obtained from a least-squares refinement of the setting angles of reflections in the range 4.453° < θ < 79.190°. A total of 3470 frames were collected using ω scans, with 1.3 second exposure time (8s for high-angle reflections), a rotation angle of 0.5° per frame, a crystal-detector distance of 34.0 mm, at T = 173(2) K.

Refinement: Disorder model was included for parts of the structure where the occupancies of each disorder component were refined through the use of a free variable. The sum of equivalent components was constrained to 1 i.e. 100%.

[(TMEDA)NaFe(C$_5$H$_{10}$N)$_3$]$_2$:

Crystal-Structure Determination. A crystal of $C_{42}H_{92}Fe_2N_{10}Na_2$ immersed in parabar oil was mounted at 173K using the X-TEMP2[3,4] cold temperature device under the microscope, carried to the diffractometer inside a dewar containing liquid nitrogen and then transferred into a stream of gaseous nitrogen (173 K). All measurements were made on a *RIGAKU XtaLAB Synergy R*, HyPix-Arc 100 area-detector diffractometer[5] using mirror optics monochromated Mo *K*α radiation (λ = 0.71073 Å). The unit cell constants and an orientation matrix for data collection were obtained from a least-squares refinement of the setting angles of reflections in the range 2.431° < θ < 32.858°. A total of 3442 frames were collected using ω scans, with 15.0 seconds exposure time, a rotation angle of 0.5° per frame, a crystal-detector distance of 43.0 mm, at T = 173.00(10) K.

Refinement: Twinning could be detected where the second component corresponds to a rotation of 4.4207 degrees around -0.50 0.76 -0.42 (reciprocal space), or -0.62 0.73 -0.29 (direct space), with a volume fractional contribution of 0.1027. The refinement was performed against the reflection file containing detwinned data of the major component only.

Table 1. Crystallographic data.

Compound	Fe_{Li}	Fe_{Na}	Fe_K	[(TMEDA)NaFe($C_5H_{10}N$)$_3$]$_2$
**CCDC Number**	2376520	2376521	2376522	2376523
**Empirical formula**	$C_{21}H_{56}FeLiN_3Si_3$	$C_{24}H_{65}FeN_4NaSi_3$	$C_{24}H_{65}FeKN_4Si_3$	$C_{42}H_{92}Fe_2N_{10}Na_2$
**Mol. Mass**	497.74	572.91	589.02	894.93
**Temperature/K**	173.01(10)	173.01(10)	173.01(10)	173.00(10)
**Crystal system**	monoclinic	monoclinic	monoclinic	triclinic
**Space group**	$P2_1/c$	$P2_1/c$	$P2_1/c$	P-1
**a/Å**	16.25953(9)	17.0909(6)	17.2884(3)	13.81828(16)
**b/Å**	10.67077(5)	11.3113(4)	11.34533(13)	15.3965(3)
**c/Å**	18.54284(9)	19.4187(5)	19.9847(3)	15.5042(2)
**α/°**	90	90	90	118.9283(17)
**β/°**	91.3100(5)	90.513(3)	97.3964(13)	95.8594(11)
**γ/°**	90	90	90	110.2937(13)
**V/Å^3**	3216.37(3)	3753.9(2)	3887.24(9)	2557.30(8)
**Z**	4	4	4	2
**λ/Å**	1.54184	1.54184	1.54184	0.71073
**2Θ range for data collection/°**	5.436 to 147.982	5.17 to 134.812	5.154 to 142.524	4.168 to 61.014
**ρ_{calc}g/cm^3**	1.028	1.014	1.006	1.162
**μ/mm^{-1}**	4.895	4.363	5.063	0.622
**F(000)**	1096.0	1264.0	1296.0	976.0
**Crystal size/mm^3**	0.231 × 0.166 × 0.139	0.067 × 0.054 × 0.018	0.19 × 0.11 × 0.07	0.201 × 0.156 × 0.129
**Reflection collected**	65002	64053	40315	97653
**Unique reflections**	6526	6747	7551	15525
***R*int**	0.0304	0.1187	0.0333	0.0437
**Goof**	1.024	1.055	1.059	1.027
**Final R indexes [I>=2σ (I)]**	R_1 = 0.0238, wR_2 = 0.0627	R_1 = 0.0479, wR_2 = 0.1241	R_1 = 0.0391, wR_2 = 0.1062	R_1 = 0.0391, wR_2 = 0.1041
**Final R indexes [all data]**	R_1 = 0.0253, wR_2 = 0.0639	R_1 = 0.0731, wR_2 = 0.1380	R_1 = 0.0479, wR_2 = 0.1121	R_1 = 0.0566, wR_2 = 0.1108
**Largest diff. peak/hole / e Å^{-3}**	0.25/-0.22	0.23/-0.48	0.33/-0.34	0.72/-0.31

Molecular structures:

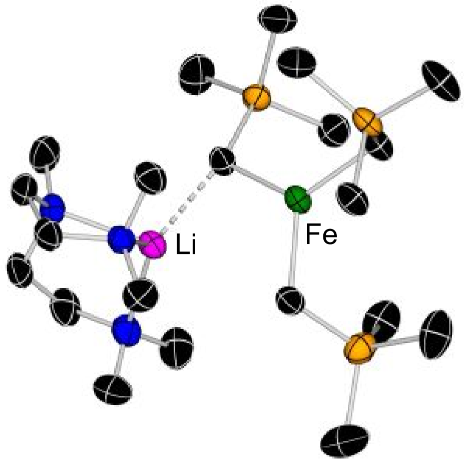

*Figure S 7. Molecular structure of (**PMDETA)LiFe(CH$_2$SiMe$_3$)$_3$**. Ellipsoids are displayed at 50% probability and all H atoms have been omitted for clarity.*

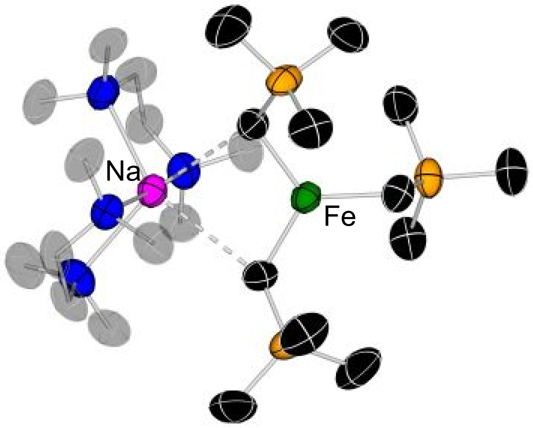

*Figure S 8. Molecular structure of (**TMEDA)$_2$NaFe(CH$_2$SiMe$_3$)$_3$**. Ellipsoids are displayed at 50% probability and all H atoms have been omitted for clarity.*

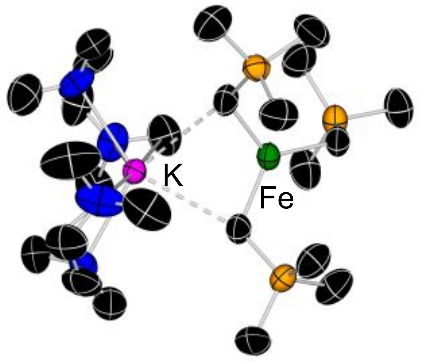

*Figure S 9. Molecular structure of (**TMEDA)$_2$KFe(CH$_2$SiMe$_3$)$_3$**. Ellipsoids are displayed at 50% probability and all H atoms have been omitted for clarity.*

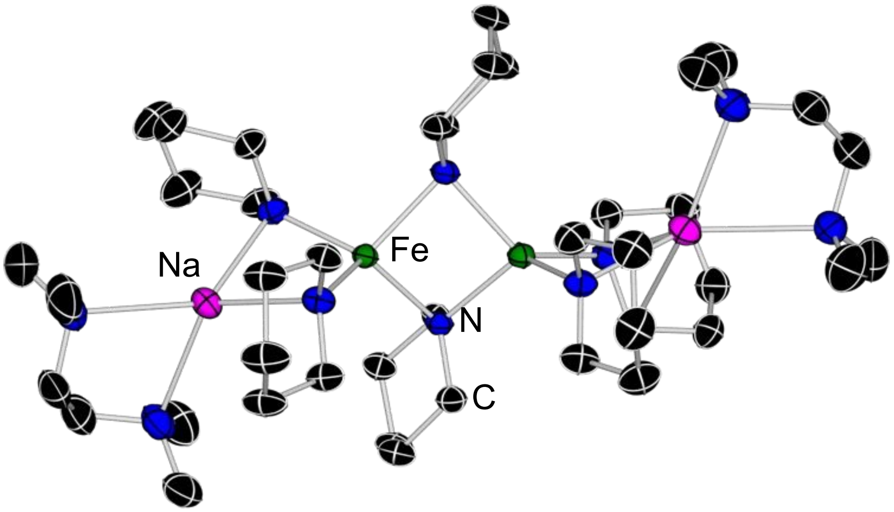

*Figure S 10. Molecular structure of **[(TMEDA)NaFe($C_5H_{10}N$)$_3$]$_2$**. Ellipsoids are displayed at 50% probability and all H atoms have been omitted for clarity.*

Stoichiometric Reaction with $NaFe(HMDS)_3$

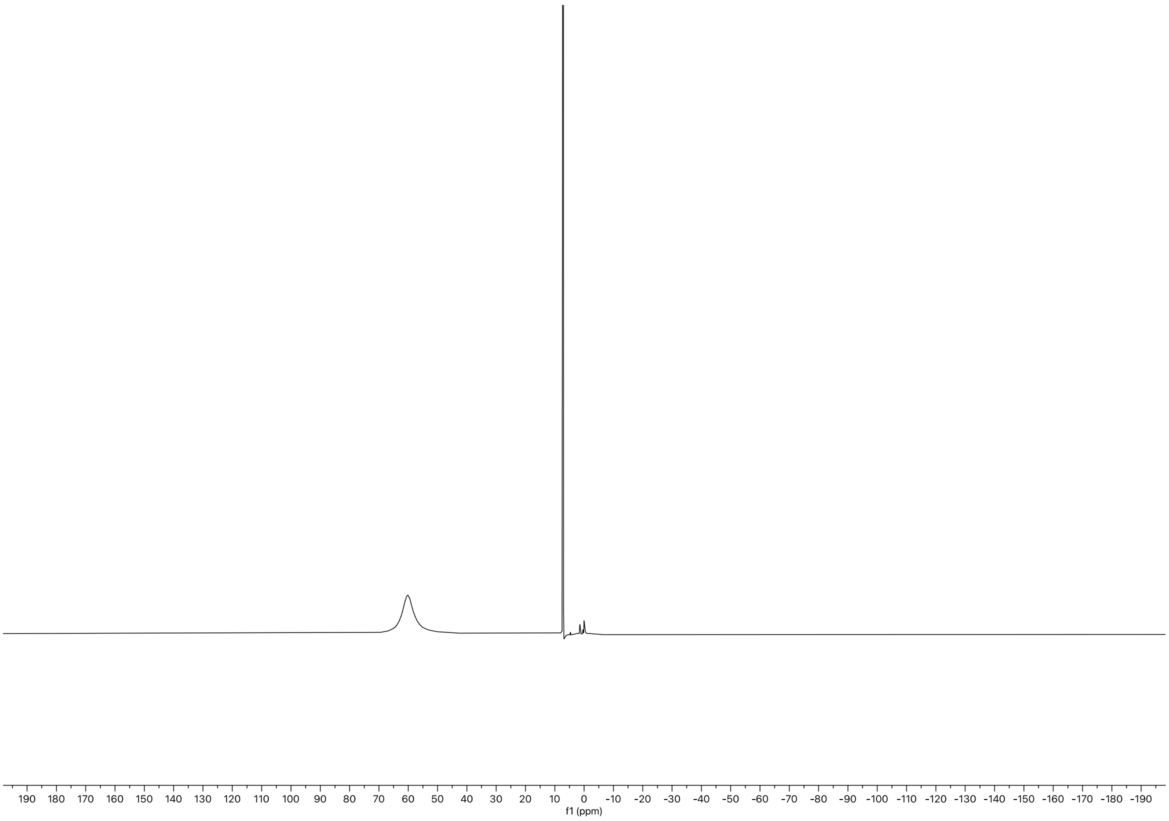

Figure S 11. ^{1}H NMR spectrum of $NaFe(HMDS)_3$ in C_6D_6..

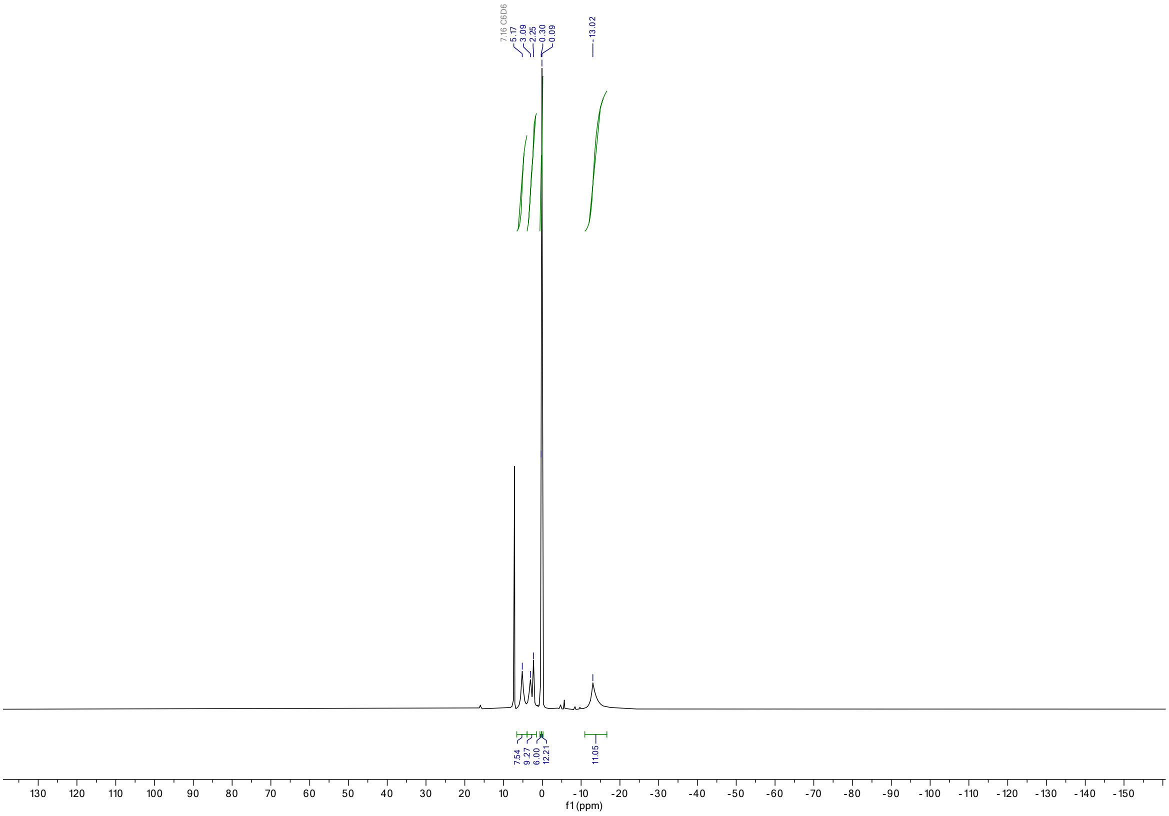

*Figure S 12. ^{1}H NMR spectrum of the reaction of $NaFe(HMDS)_3$ with 3 equivalents of piperidine in C_6D_6. Full conversion of $NaFe(HMDS)_3$ with the formation of 2 equivalents of HMDS(H) is observed. Paramagnetic signals are different from the ones observed for **I**, suggesting that the transamidation is not complete.*

Catalytic Reactions

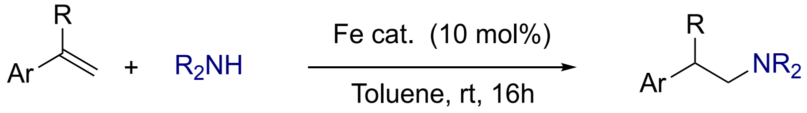

Typical Procedure

In a J-young NMR tube or a GC-vial in the glovebox, the catalyst was added (0.02 mmol, 10 mol%) and it was dissolved in toluene (or THF). The amine (0.25 mmol) and the vinylarene (0.2 mmol) were then added and the reaction was left at room temperature for 16 hours. After that time, the reaction vessel was opened to air, diluted with EtOAc and filtered through silica gel. The organic solvent was removed under reduced pressure to give the crude compounds.

- For figure 2: hexamethylbenzene was added as internal standard and an assay yield was calculated by ^{1}H NMR spectroscopy.
- For isolated yields: the compounds were transferred into a vial and dried under high vacuum, or alternatively they were further purified by column chromatography.

-Reaction with Fe_{Li} (10 mol%, 10.0 mg): 89% yield

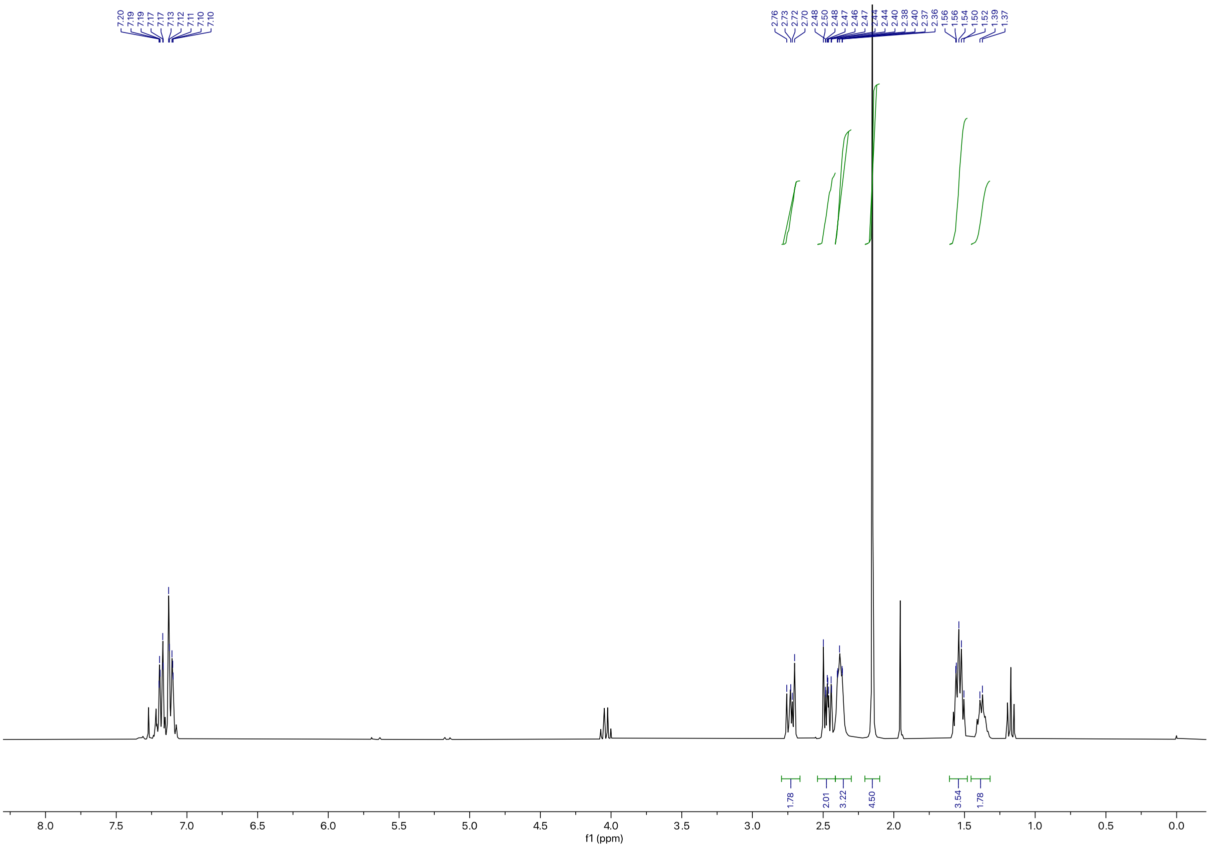

Figure S 13. ^{1}H NMR spectrum of the reaction with Fe_{Li}, using C_6Me_6 (0.1 mmol) as internal standard.

-Reaction with Fe_{Na} (10 mol%, 11.4 mg): 98% yield

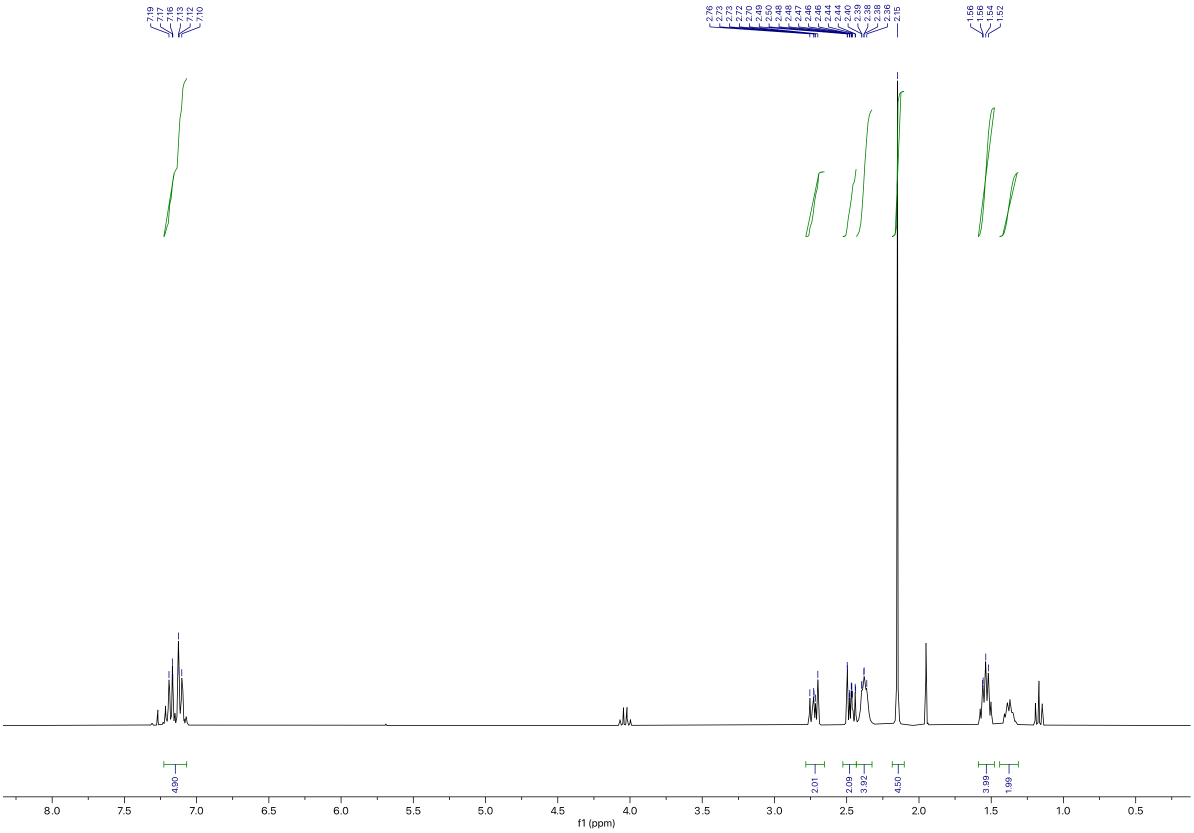

Figure S 14. ^{1}H NMR spectrum of the reaction with Fe_{Na}, using C_6Me_6 (0.1 mmol) as internal standard.

-Reaction with Fe_K (10 mol%, 11.8 mg): 34% yield

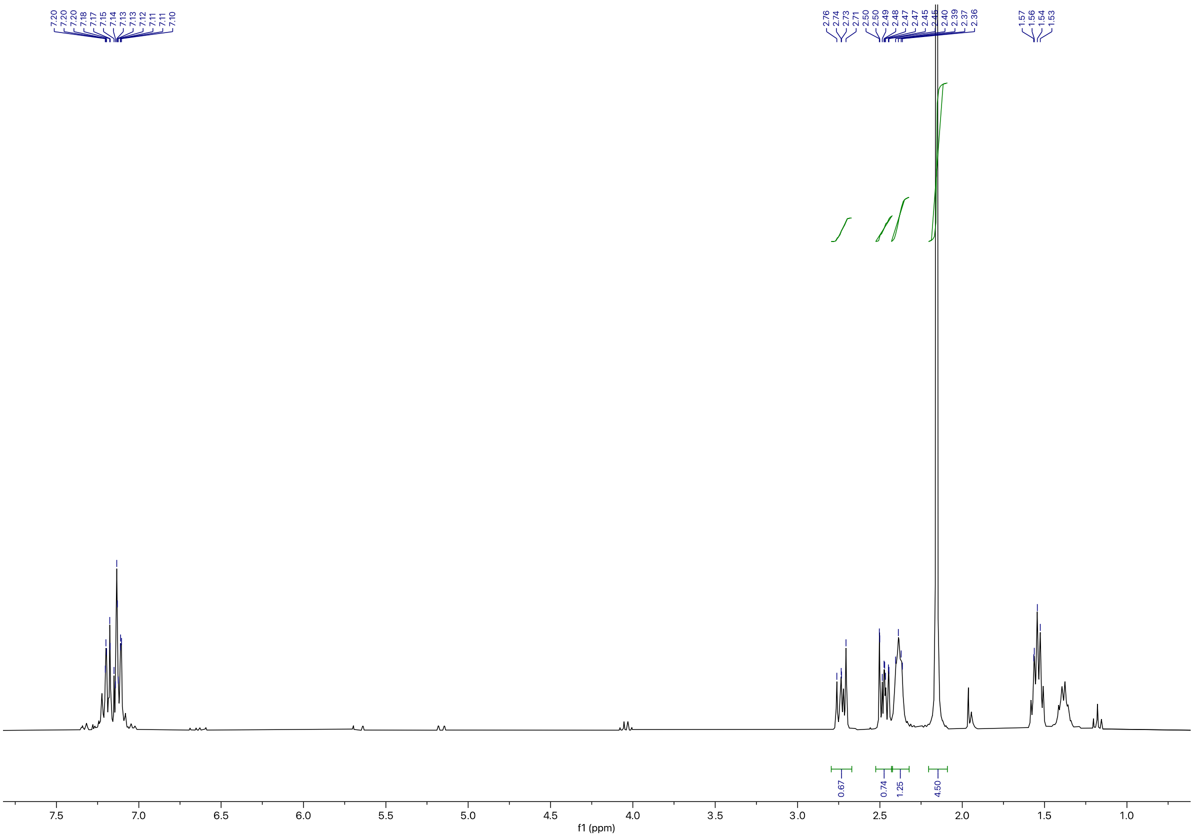

Figure S 15. ^{1}H NMR spectrum of the reaction with Fe_K, using C_6Me_6 (0.1 mmol) as internal standard.

-Reaction with $NaCH_2SiMe_3$ (10 mol%, 2.2mg) and TMEDA (10 mol%, 3 μL): 60%yield

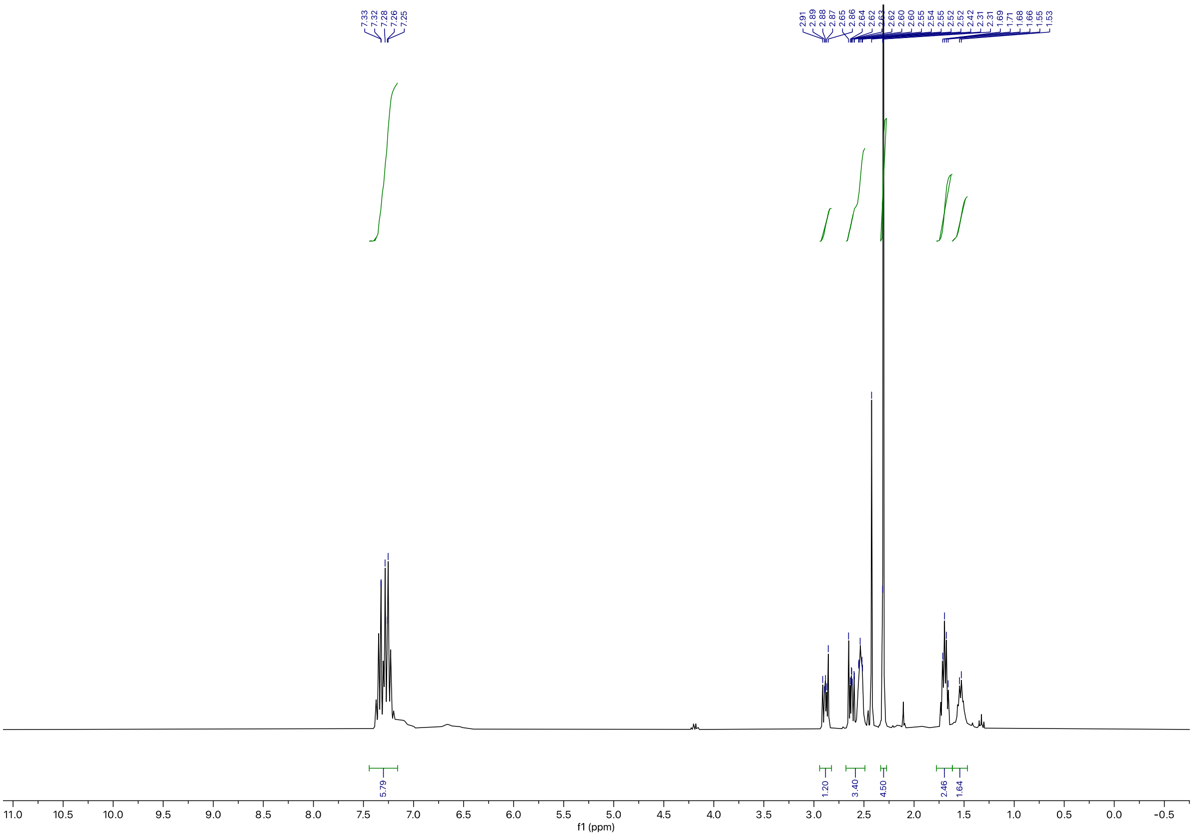

Figure S 16. ^1H NMR spectrum of the reaction with $NaCH_2SiMe_3$, using C_6Me_6 (0.1 mmol) as internal standard.

-Reaction with Fe_{Na} (10 mol%, 11.4 mg) in THF at 50 ºC: 94% yield.

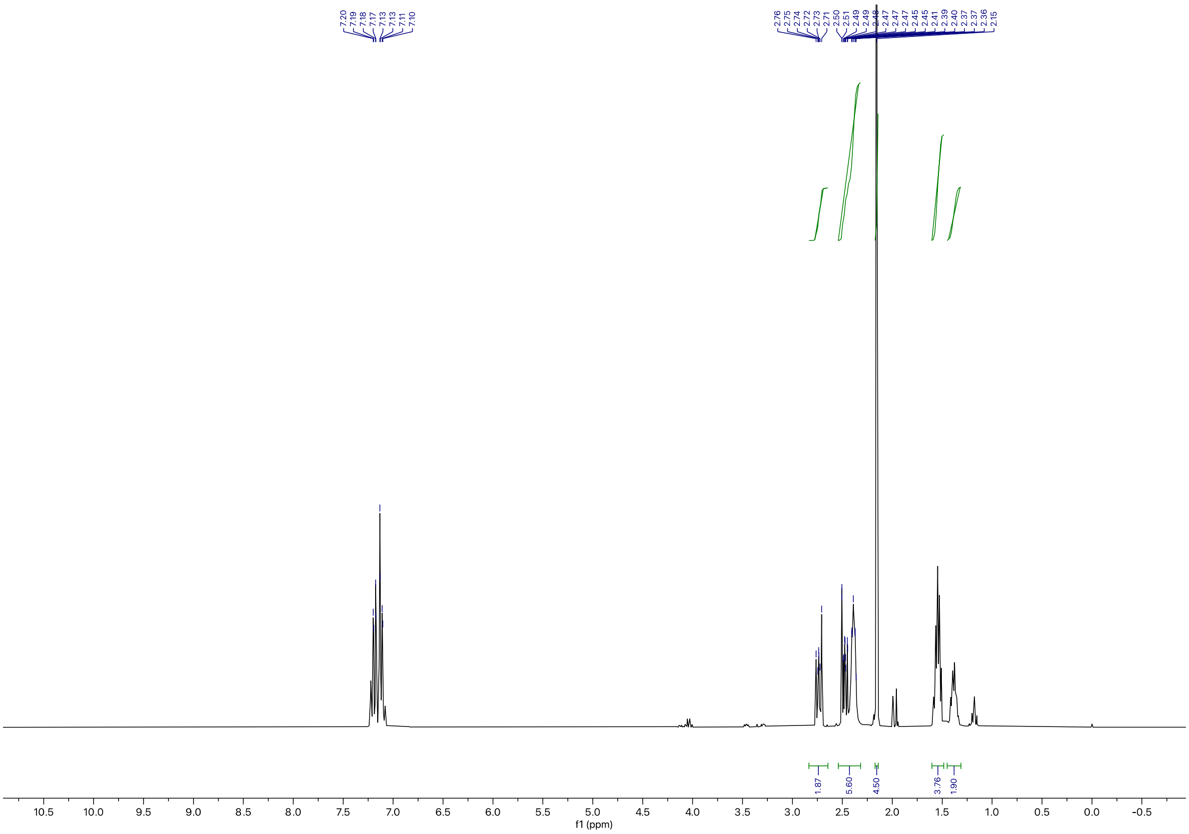

Figure S 17. ^{1}H NMR spectrum of the reaction with Fe_{Na} in THF at 50 ºC, using C_6Me_6 (0.1 mmol) as internal standard.

-Reaction with Fe_{Na} (5 mol%, 5.7 mg): 16% yield

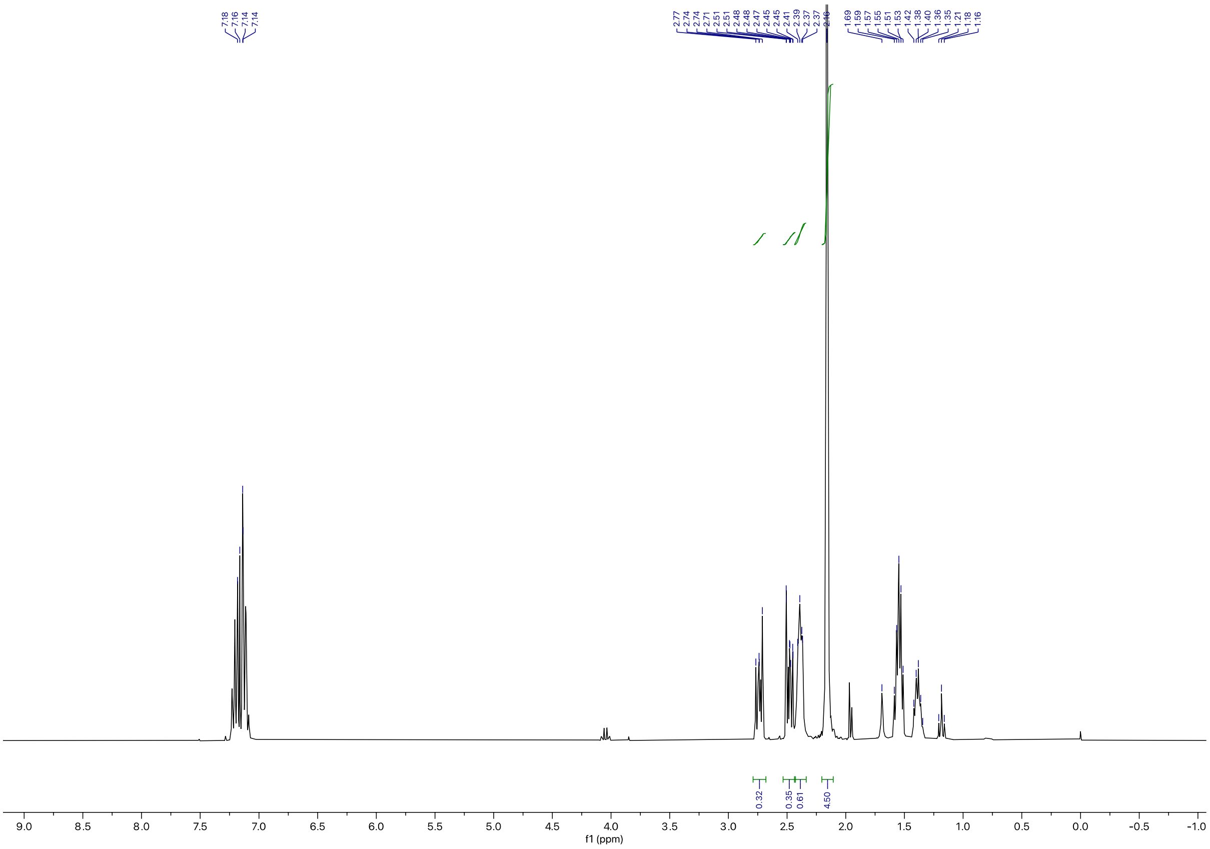

Figure S 18. ^{1}H NMR spectrum of the reaction with Fe_{Na}, using C_6Me_6 (0.1 mmol) as internal standard.

-Reaction with **I** (10 mol%, 8.9 mg): 93% yield

In an NMR tube, piperidine (20 μL; 0.2 mmol) was dissolved in THF. Compound **I** was added (8.9 mg, 10 mol%) followed by styrene (22.8 μL; 0.20 mmol) and toluene. The reaction was left at room temperature for 16 hours. After that time, the reaction was opened to air, diluted with EtOAc and filtered through silica gel to give compound **1a** as a pure oil (35.2mg, 93% yield).

Cross-over Experiment with I and morpholine

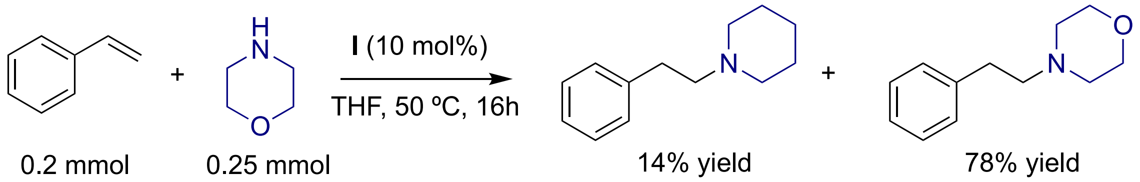

In an NMR tube, morpholine (22 μL; 0.25 mmol) was dissolved in THF. Compound **I** was added (8.9 mg, 10 mol%) followed by styrene (22.8 μL; 0.20 mmol). The reaction was heated at 50 ºC for 16 hours. After that time, the reaction was opened to air, diluted with EtOAc and filtered through silica gel. C_6Me_6 was added as internal standard and a ^{1}H NMR spectrum was recorded to determine the yield of the reaction.

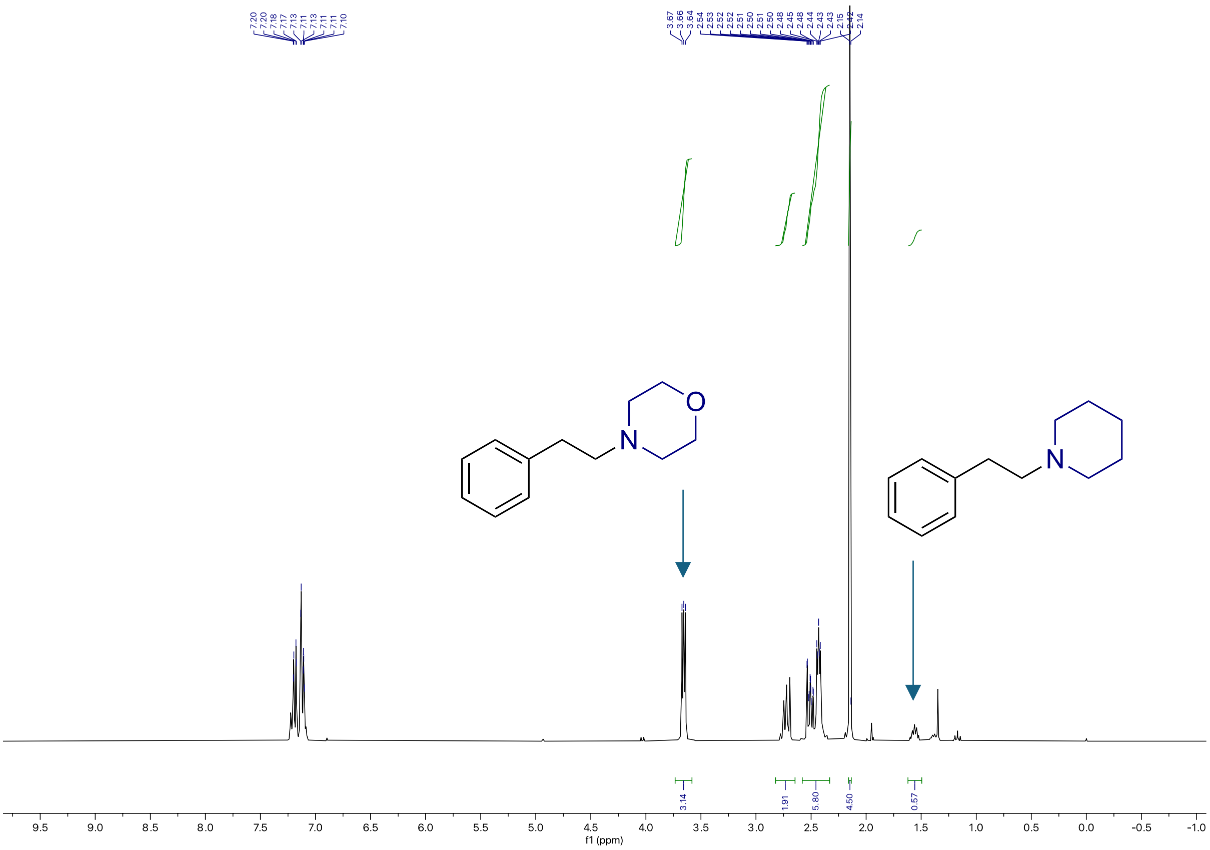

*Figure S 19. ^{1}H NMR spectrum of the cross-over experiment with **I**, using C_6Me_6 (0.1 mmol) as internal standard.*

Characterization of the products

1-phenethylpiperidine (1a). Following the general procedure using Fe_{Na} (11.4 mg, 10 mol%), styrene (22.8 μL, 0.2 mmol) and piperidine (25 μL, 0.25 mmol) the title product was obtained as a colourless oil (36.2 mg, 96% yield).
Spectroscopic data is in agreement with the literature.[9]
^{1}H NMR (300 MHz, $CDCl_3$) δ 7.33 – 7.24 (m, 2H), 7.23 – 7.17 (m, 3H), 2.89 – 2.75 (m, 2H), 2.62 – 2.51 (m, 2H), 2.51 – 2.40 (m, 4H), 1.63 (p, *J* = 5.6 Hz, 4H), 1.56 – 1.39 (m, 2H).
^{13}C{^{1}H} NMR (75 MHz, $CDCl_3$) δ 140.8, 128.9, 128.49, 126.1, 61.6, 54.7, 33.8, 26.1, 24.6.

1-(2-([1,1'-biphenyl]-4-yl)ethyl)piperidine (1b). Following the general procedure using Fe_{Na} (11.4 mg, 10 mol%), 4-phenylstyrene (36.05 mg, 0.2 mmol) and piperidine (25 μL, 0.25 mmol) the title product was obtained as a colourless oil (45.1 mg, 85% yield).
Spectroscopic data is in agreement with the literature.[9]
^{1}H NMR (300 MHz, $CDCl_3$) δ 7.55 – 7.40 (m, 4H), 7.37 – 7.28 (m, 2H), 7.28 – 7.12 (m, 3H), 2.83 – 2.72 (m, 2H), 2.57 – 2.46 (m, 2H), 2.46 – 2.36 (m, 4H), 1.62 – 1.49 (m, 4H), 1.45 – 1.31 (m, 2H).
^{13}C{^{1}H} NMR (75 MHz, $CDCl_3$) δ 141.2, 139.9, 139.1, 129.2, 128.8, 127.2, 127.1, 127.1, 61.5, 54.7, 33.4, 26.1, 24.6.

1-(4-(*tert*-butyl)phenethyl)piperidine (1c). Following the general procedure using Fe_{Na} (11.4 mg, 10 mol%), 4-tertbutylstyrene (36.6 μL, 0.2 mmol) and piperidine (25 μL, 0.25 mmol) the title product was obtained as a colourless oil (48.6 mg, 97% yield).
Spectroscopic data is in agreement with the literature. [10]
^{1}H NMR (300 MHz, $CDCl_3$) δ 7.33 – 7.17 (m, 2H), 7.06 (d, *J* = 8.6 Hz, 2H), 2.76 – 2.64 (m, 2H), 2.55 – 2.43 (m, 2H), 2.44 – 2.33 (m, 4H), 1.64 – 1.45 (m, 4H), 1.44 – 1.30 (m, 2H), 1.22 (s, 9H).
^{13}C{^{1}H} NMR (75 MHz, $CDCl_3$) δ 148.8, 137.6, 128.5, 125.3, 61.6, 54.7, 34.4, 33.2, 31.5, 26.1, 24.6.

1-(4-methylphenethyl)piperidine (1d). Following the general procedure using Fe_{Na} (11.4 mg, 10 mol%), 4-methylstyrene (25.9 μL, 0.2 mmol) and piperidine (25 μL, 0.25 mmol) the title product was obtained as a colourless oil (40.5 mg, 98% yield).
Spectroscopic data is in agreement with the literature. [9]
^{1}H NMR (300 MHz, $CDCl_3$) δ 7.01 (s, 4H), 2.78 – 2.58 (m, 2H), 2.52 – 2.34 (m, 6H), 2.23 (s, 3H), 1.61 – 1.48 (m, 4H), 1.44 – 1.30 (m, 2H).
^{13}C{^{1}H} NMR (75 MHz, $CDCl_3$) δ 137.7, 135.5, 129.1, 128.7, 61.7, 54.7, 33.3, 26.1, 24.6, 21.1.

1-(3-fluorophenethyl)piperidine (1e). Following the general procedure using Fe_{Na} (11.4 mg, 10 mol%), 3-fluorostyrene (24 μL, 0.2 mmol) and piperidine (25 μL, 0.25 mmol) the title product was obtained as a colourless oil (13.5 mg, 32% yield).
Spectroscopic data is in agreement with the literature. [10]
^{1}H NMR (300 MHz, $CDCl_3$) δ 7.27 – 7.16 (m, 1H), 7.04 – 6.78 (m, 3H), 2.92 – 2.73 (m, 2H), 2.63 – 2.42 (m, 6H), 1.73 – 1.56 (m, 4H), 1.56 – 1.40 (m, 2H).
^{13}C{^{1}H} NMR (75 MHz, $CDCl_3$) δ 163.0 (d, *J* = 245.2 Hz), 129.9 (d, *J* = 8.3 Hz), 124.5 (d, *J* = 2.8 Hz), 115.7 (d, *J* = 20.7 Hz), 113.0 (d, *J* = 21.0 Hz), 61.0, 54.7, 33.5 (d, *J* = 1.7 Hz), 26.1, 24.5.
^{19}F NMR (282 MHz, $CDCl_3$) δ -113.76.

1-(2,2-diphenylethyl)piperidine (1f). Following the general procedure using Fe_{Na} (11.4 mg, 10 mol%), 1,1-diiphenylethylene (35.3 μL, 0.2 mmol) and piperidine (25 μL, 0.25 mmol) the title product was obtained as a colourless oil (39.1 mg, 74% yield).
Spectroscopic data is in agreement with the literature. [9]
^{1}H NMR (300 MHz, $CDCl_3$) δ 7.21 – 7.03 (m, 10H), 4.15 (t, *J* = 7.3 Hz, 1H), 2.87 (d, *J* = 7.2 Hz, 2H), 2.32 (t, *J* = 5.4 Hz, 4H), 1.45 – 1.35 (m, 4H), 1.35 – 1.24 (m, 2H).
^{13}C{^{1}H} NMR (75 MHz, $CDCl_3$) δ 144.5, 128.4, 128.3, 126.2, 64.6, 55.0, 49.0, 26.1, 24.5.

4-phenethylmorpholine (1g). Following the general procedure using Fe_{Na} (11.4 mg, 10 mol%), styrene (22.8 μL, 0.2 mmol) and morpholine (22 μL, 0.25 mmol) the title product was obtained as a colourless oil (39.3 mg, 95% yield).
Spectroscopic data is in agreement with the literature. [10]
^{1}H NMR (300 MHz, $CDCl_3$) δ 7.34 – 7.17 (m, 5H), 3.87 – 3.44 (m, 4H), 2.93 – 2.71 (m, 2H), 2.66 – 2.57 (m, 2H), 2.57 – 2.49 (m, 4H).
^{13}C{^{1}H} NMR (75 MHz, $CDCl_3$) δ 140.2, 128.8, 128.5, 126.2, 67.1, 61.0, 53.8, 33.4.

1-methyl-4-phenethylpiperazine (1h). Following the general procedure using Fe_{Na} (11.4 mg, 10 mol%), styrene (22.8 μL, 0.2 mmol) and *N*-methylpiperazine (27.7 μL, 0.25 mmol) the title product was obtained as a colourless oil (31.2 mg, 76% yield).
Spectroscopic data is in agreement with the literature. [10]
^{1}H NMR (300 MHz, $CDCl_3$) δ 7.37 – 7.13 (m, 5H), 2.94 – 2.72 (m, 2H), 2.73 – 2.38 (m, 10H), 2.31 (s, 3H).
^{13}C{^{1}H} NMR (75 MHz, $CDCl_3$) δ 140.4, 128.8, 128.4, 126.1, 60.6, 55.2, 53.2, 46.1, 33.7.

***N*,*N*-dibenzyl-2-phenylethan-1-amine (1i).** Following the general procedure using Fe_{Na} (11.4 mg, 10 mol%), styrene (22.8 μL, 0.2 mmol) and dibenzylamine (27.7 μL, 0.25 mmol) the title product was obtained as a colourless oil. Hexamethylbenzene was added as internal standard, and the yield was calculated by ^{1}H NMR spectroscopy to be 68%.
Spectroscopic data is in agreement with the literature. [10]

^{1}H NMR (300 MHz, $CDCl_3$) δ 7.42 – 7.01 (m, 15H), 3.65 (s, 4H), 2.86 – 2.77 (m, 2H), 2.76 – 2.67 (m, 2H).

Copies of NMR Spectra

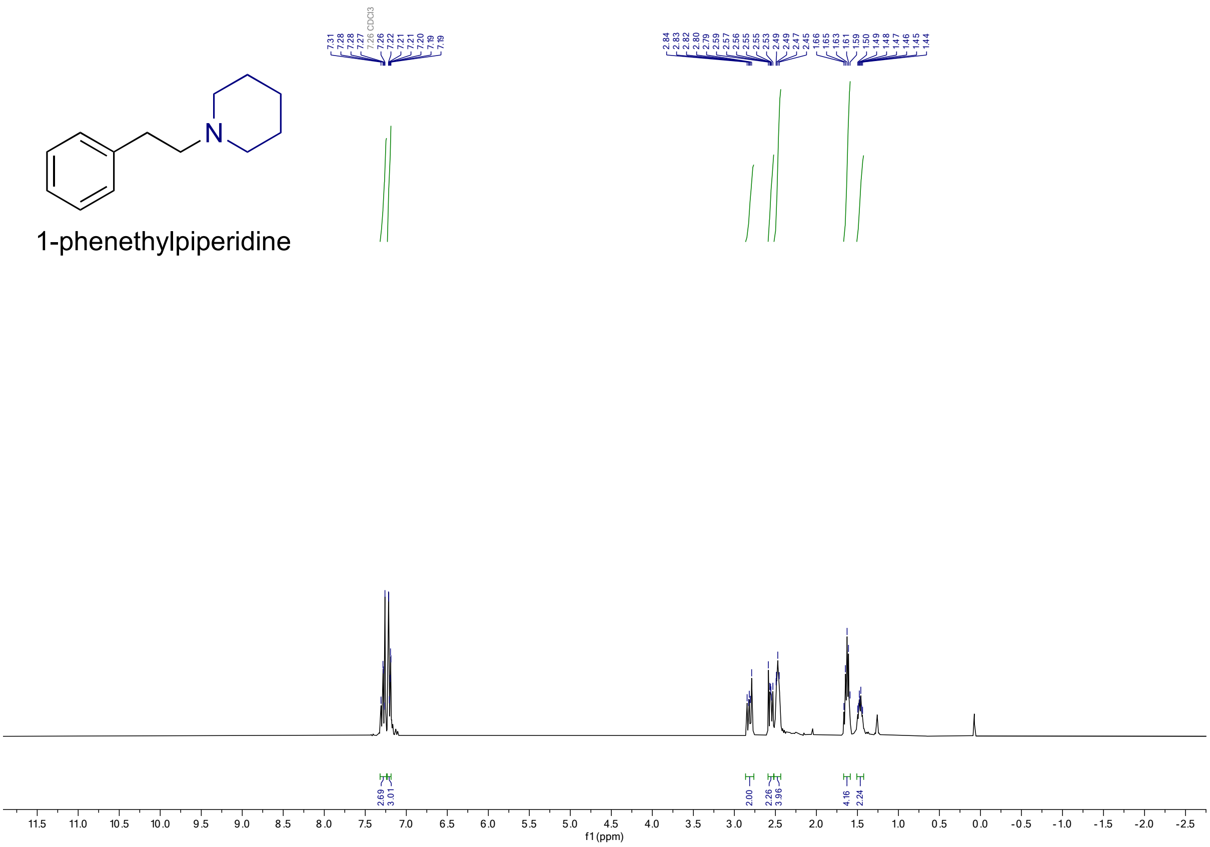

*Figure S 20. ^{1}H NMR spectrum of **1a** in $CDCl_3$ (Spectrometer: 300 MHz).*

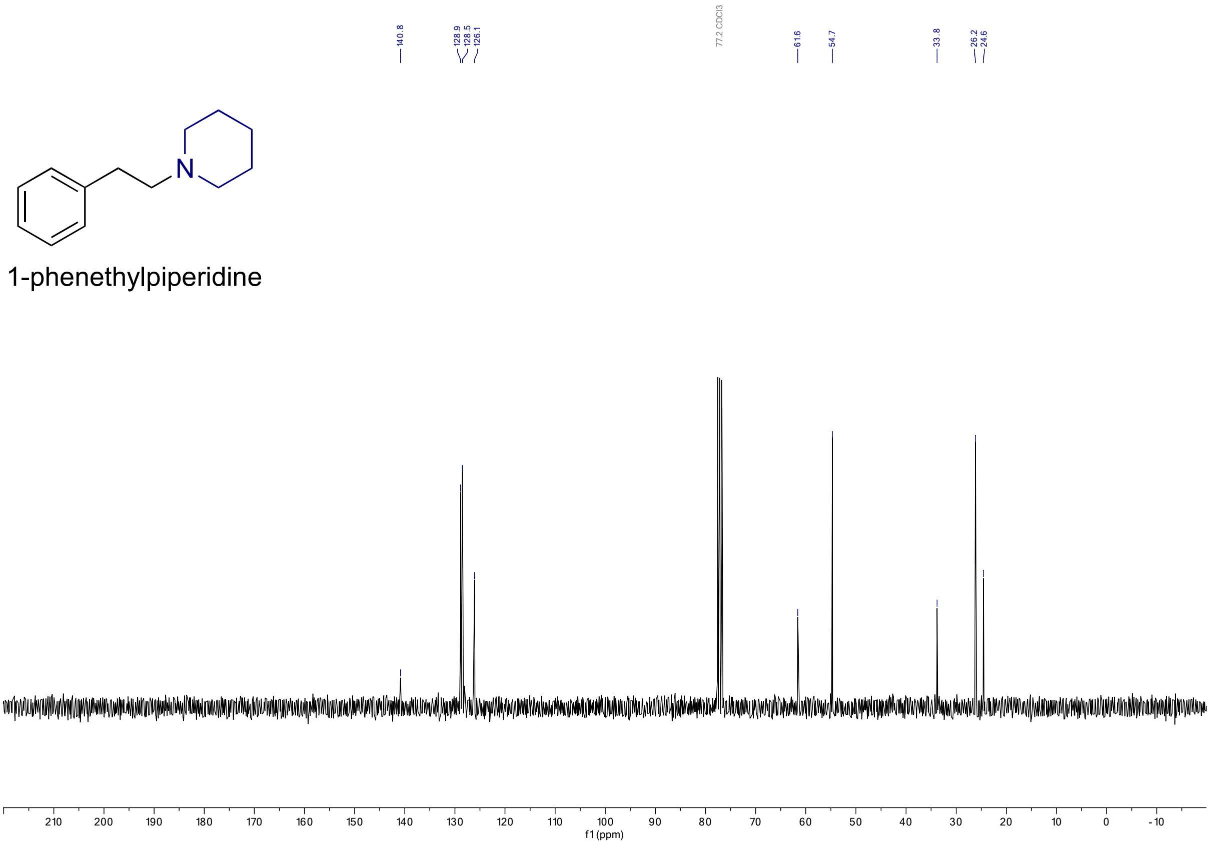

*Figure S 21. $^{13}C\{^{1}H\}$ NMR spectrum of **1a** in $CDCl_3$ (Spectrometer: 300 MHz).*

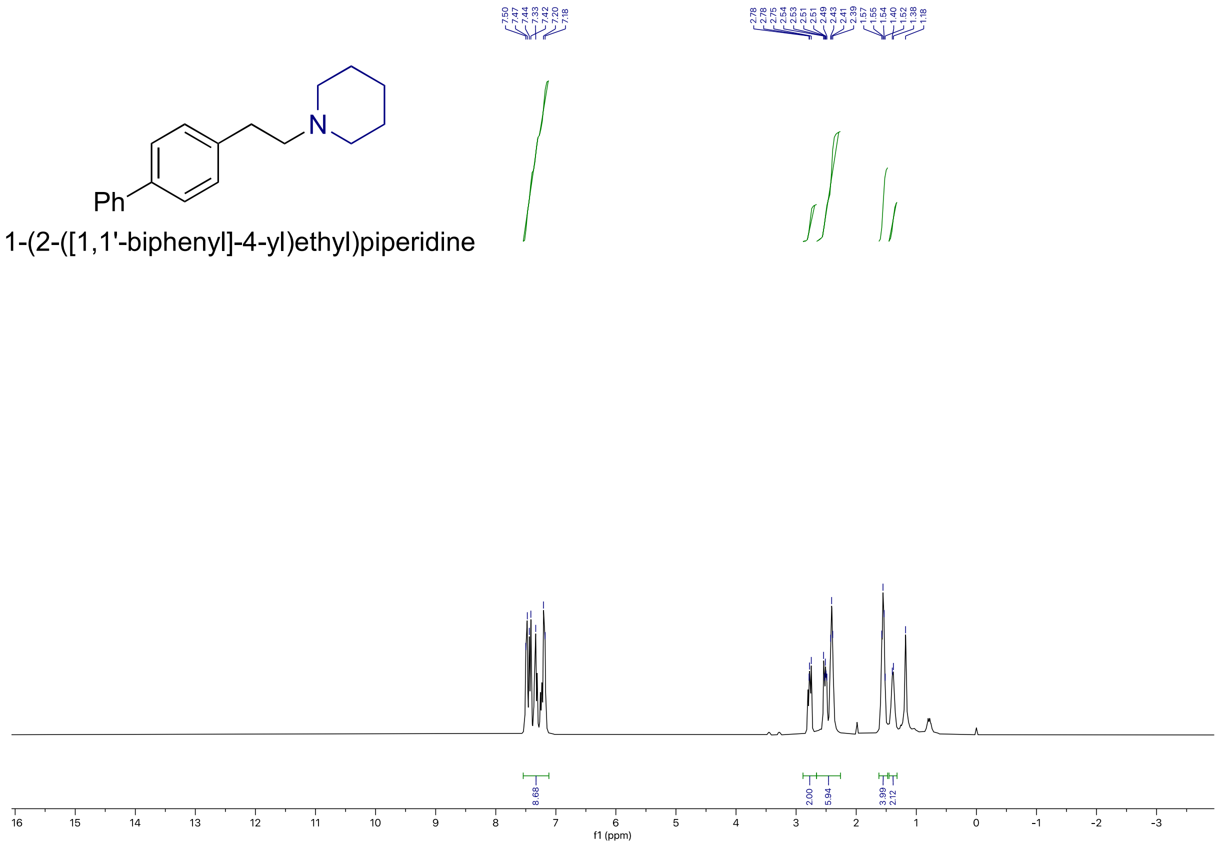

Figure S 22. ^{1}H NMR spectrum of ***1b*** *in $CDCl_3$ (Spectrometer: 300 MHz).*

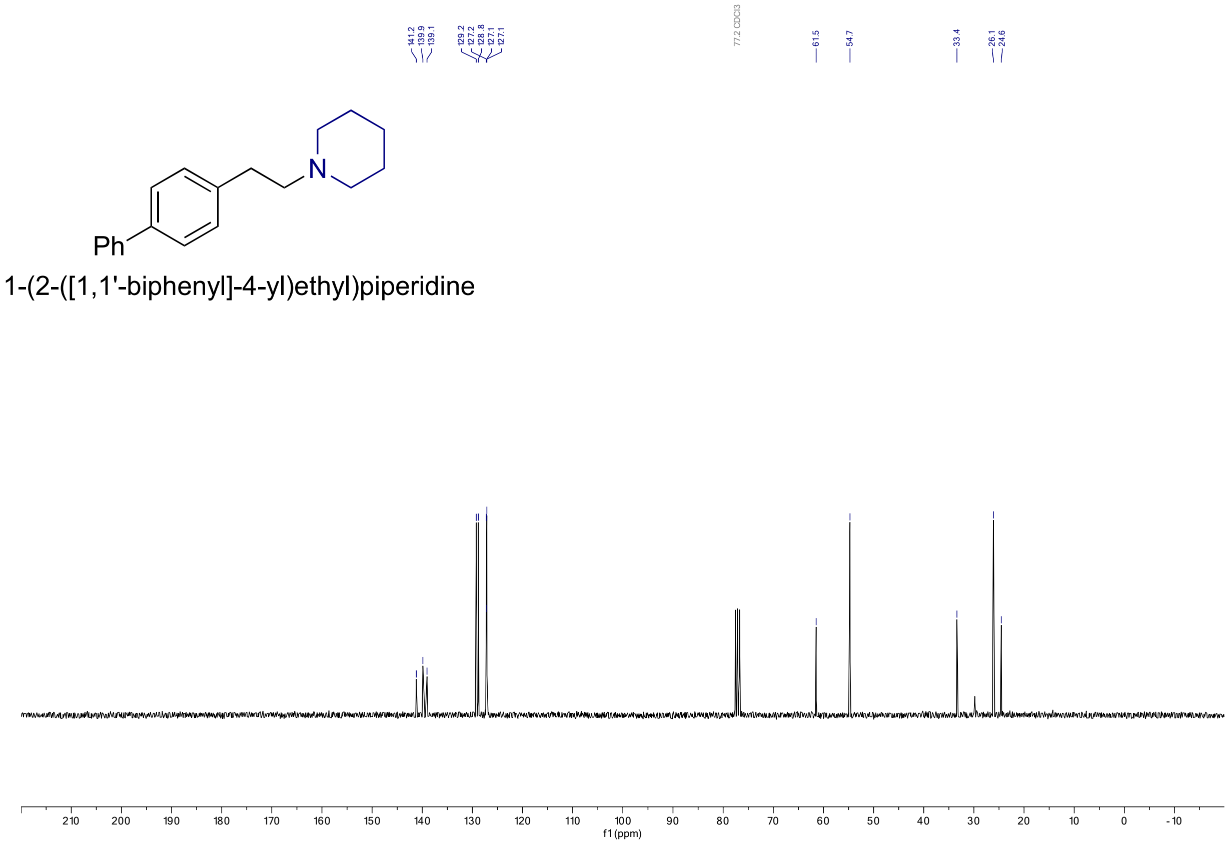

Figure S 23. $^{13}C\{^{1}H\}$ NMR spectrum of ***1b*** *in $CDCl_3$ (Spectrometer: 300 MHz).*

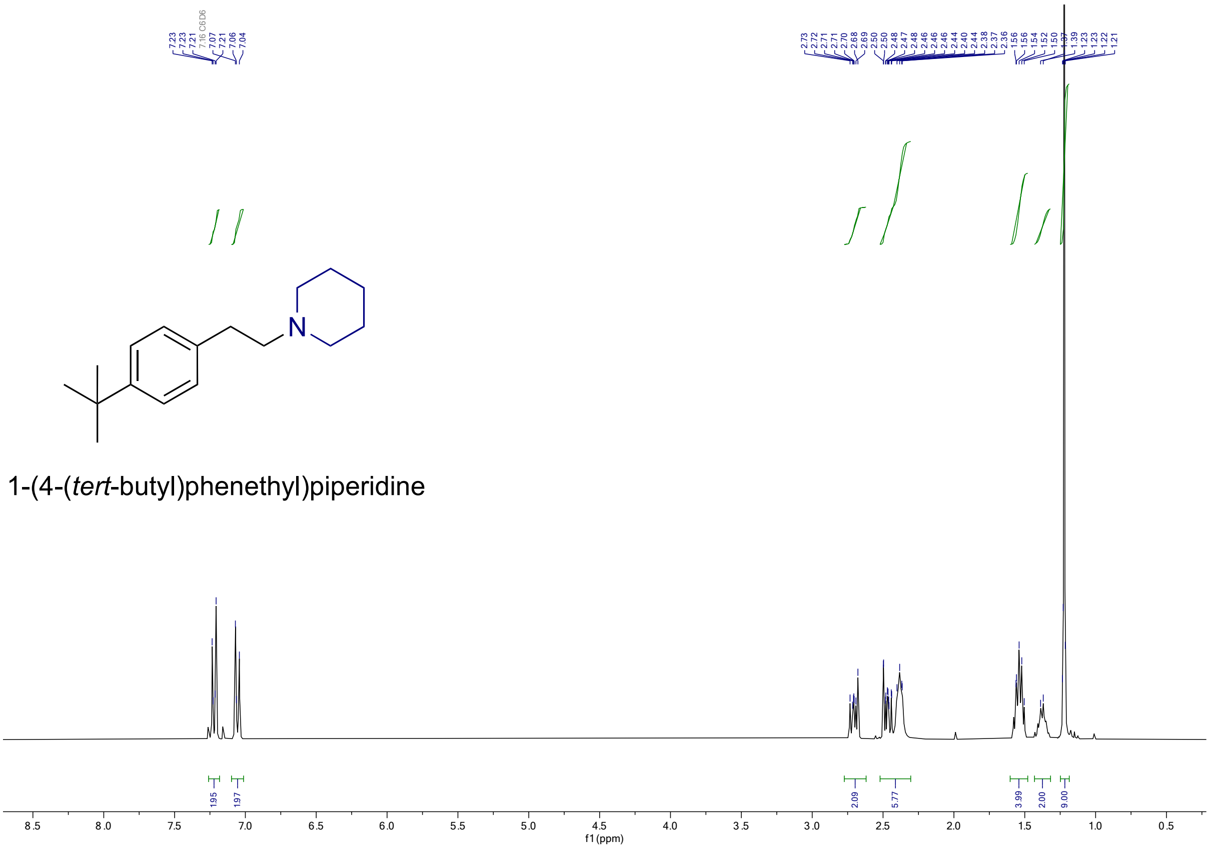

Figure S 24. ^{1}H NMR spectrum of ***1c*** *in $CDCl_3$ (Spectrometer: 300 MHz).*

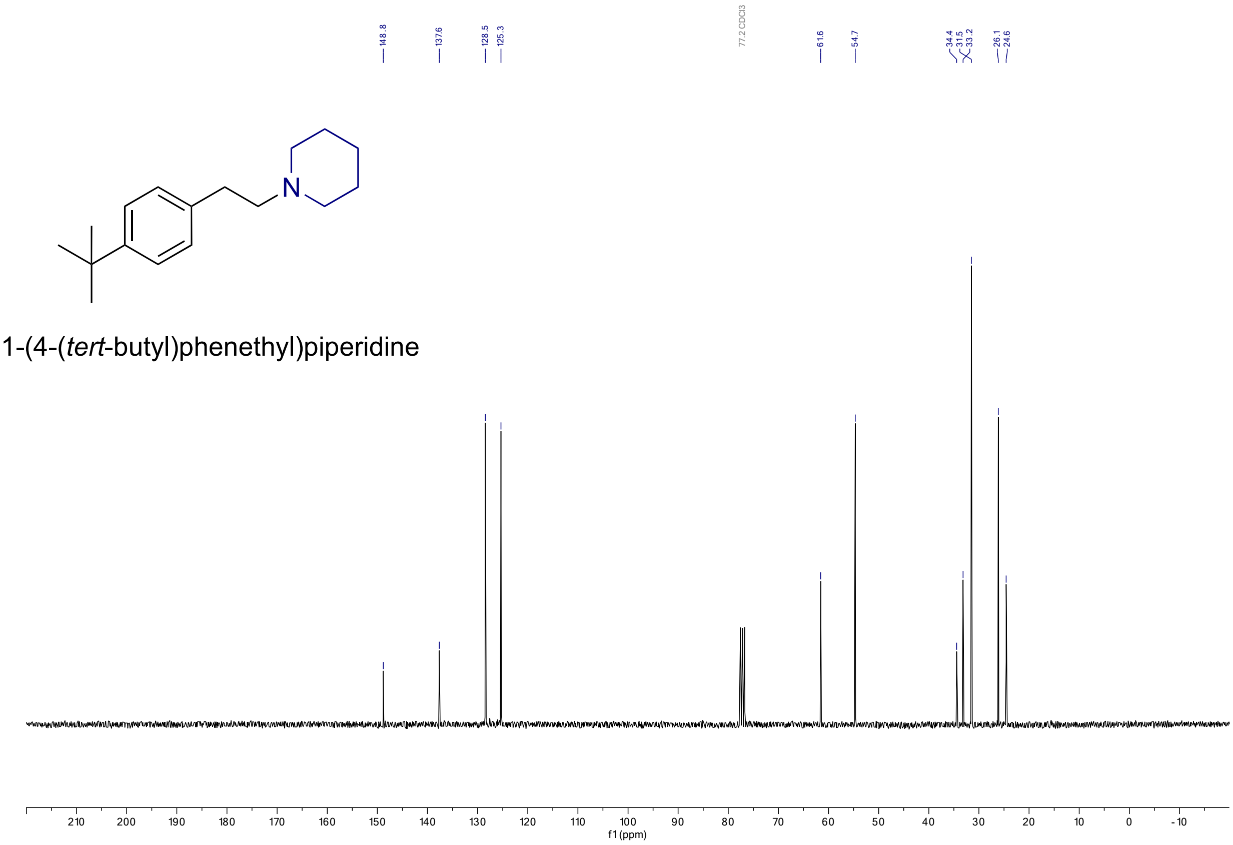

Figure S 25. $^{13}C\{^{1}H\}$ NMR spectrum of ***1c*** *in $CDCl_3$ (Spectrometer: 300 MHz).*

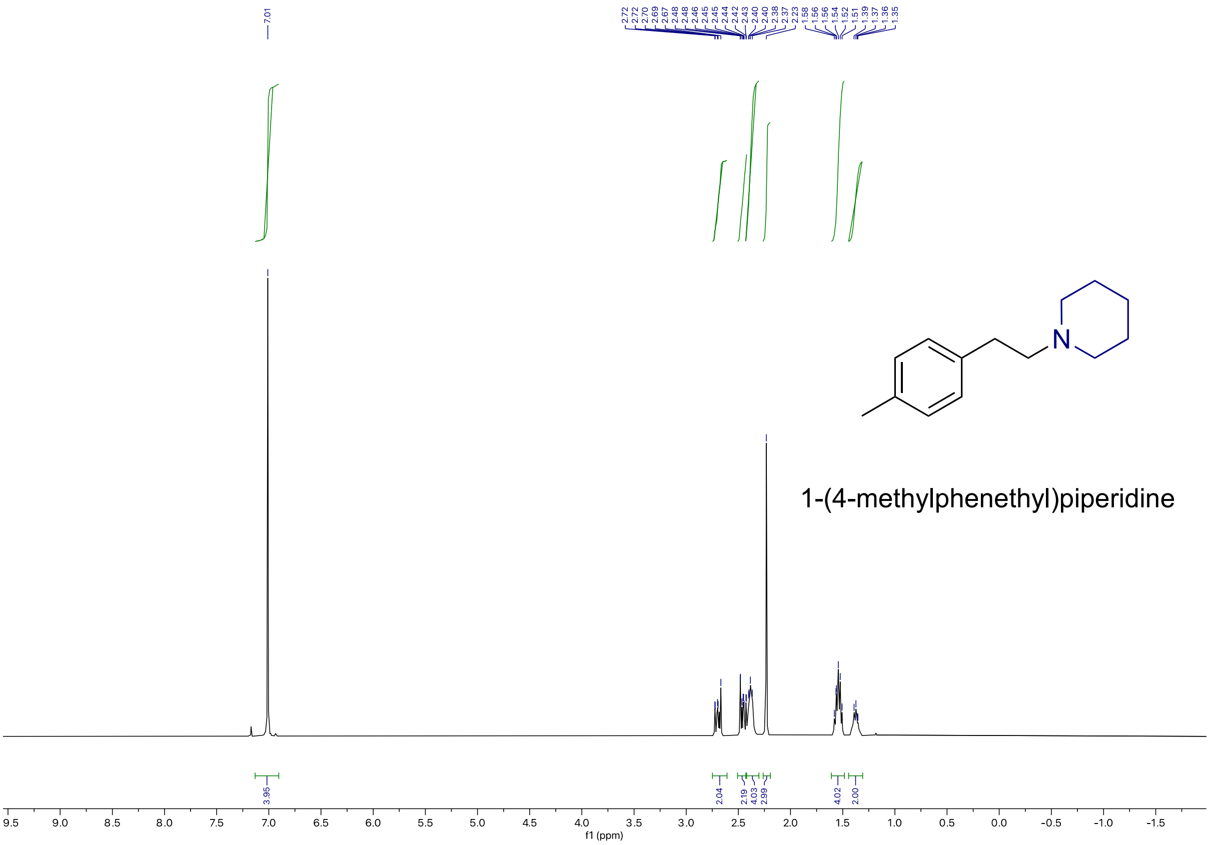

Figure S 26. ^{1}H NMR spectrum of ***1d*** *in $CDCl_3$ (Spectrometer: 300 MHz).*

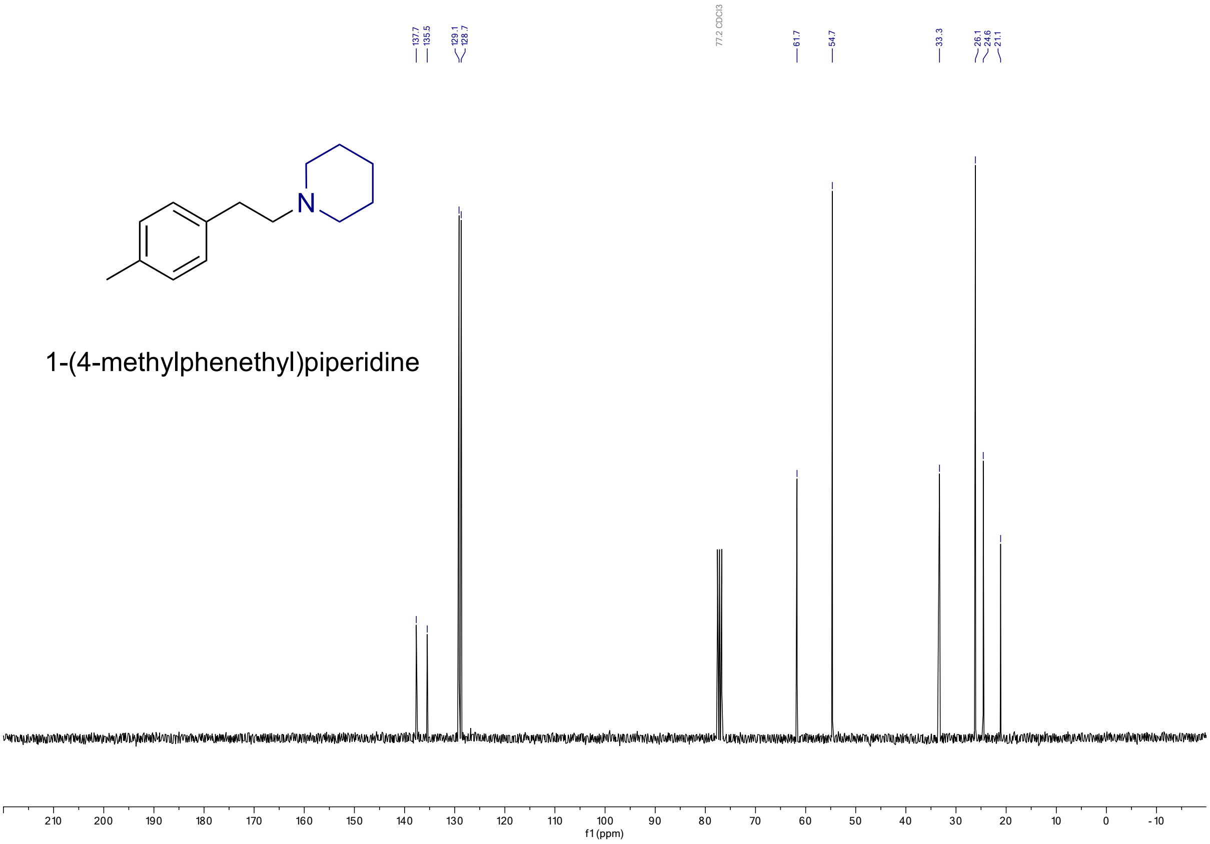

Figure S 27. $^{13}C\{^{1}H\}$ NMR spectrum of ***1d*** *in $CDCl_3$ (Spectrometer: 300 MHz).*

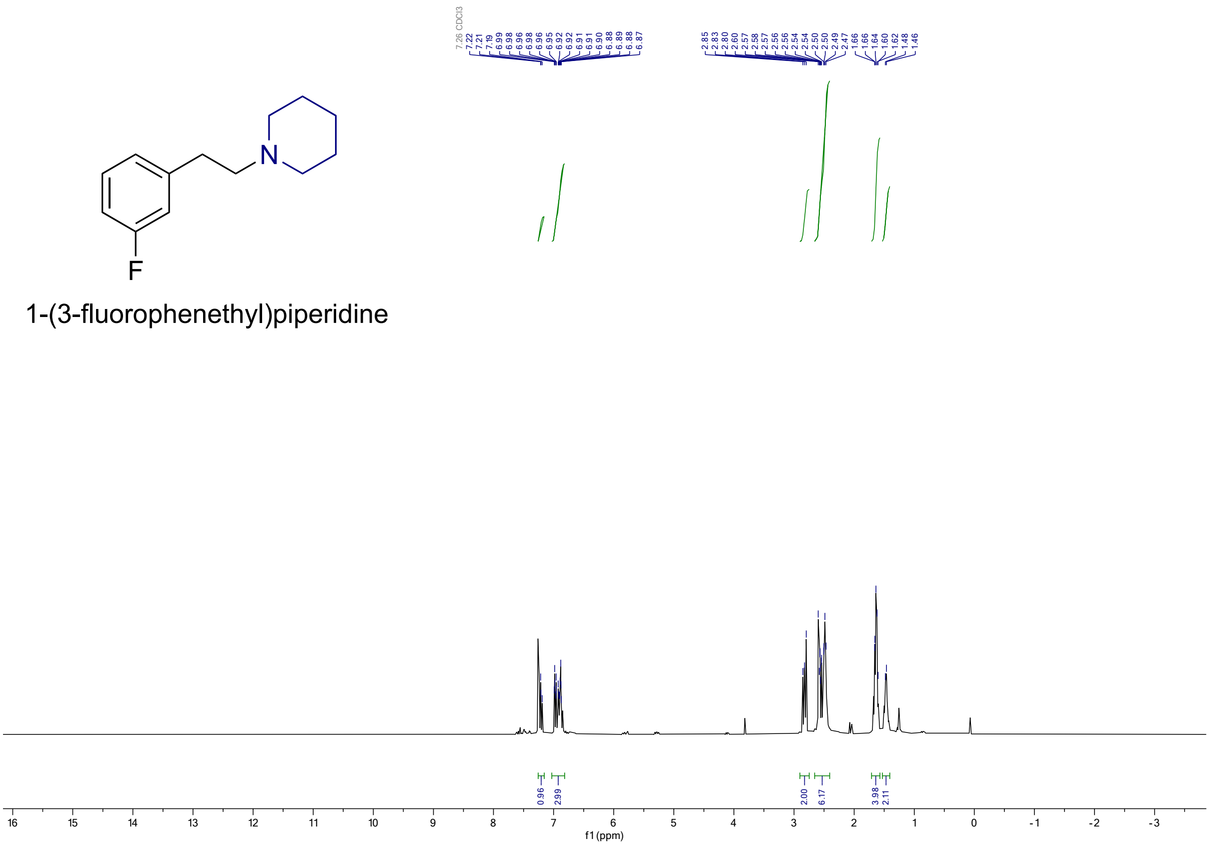

Figure S 28. ^{1}H NMR spectrum of ***1e*** *in $CDCl_3$ (Spectrometer: 300 MHz).*

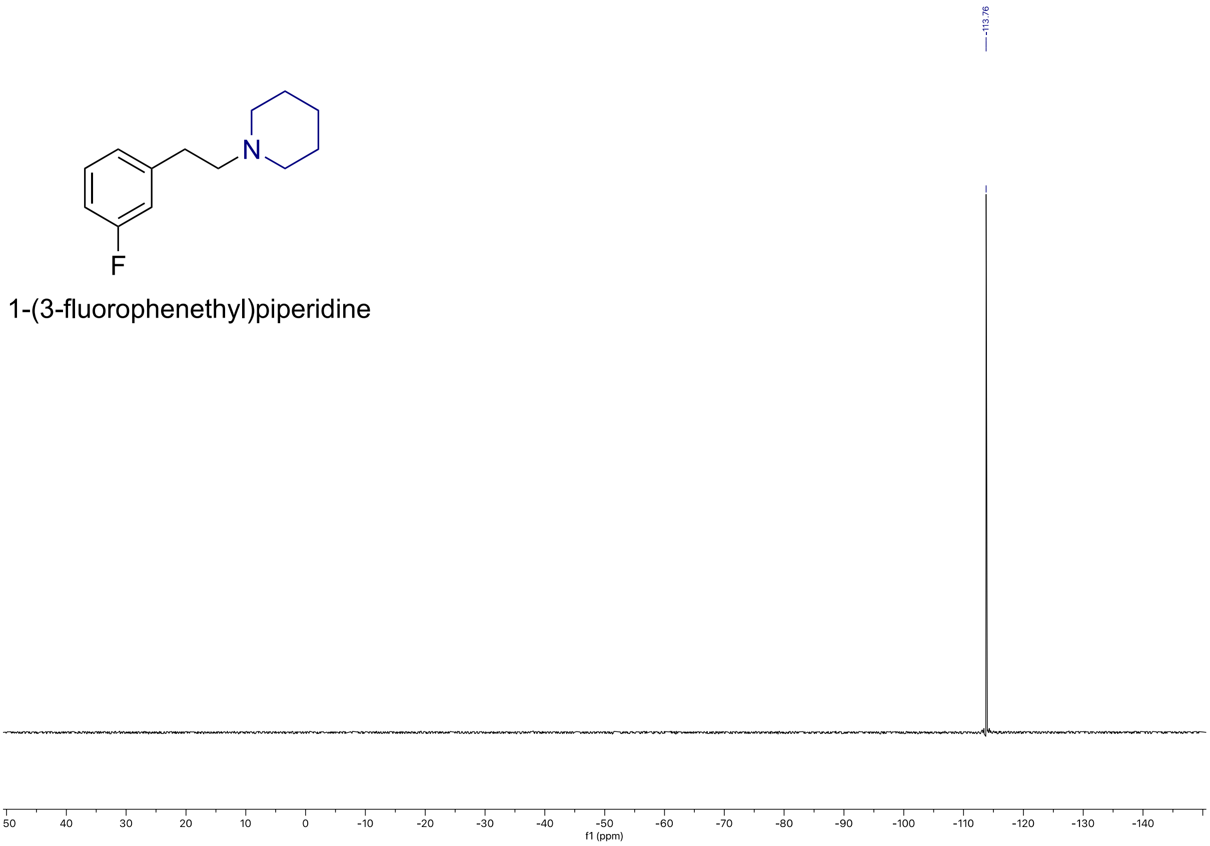

Figure S 29. ^{19}F NMR spectrum of ***1e*** *in $CDCl_3$ (Spectrometer: 300 MHz).*

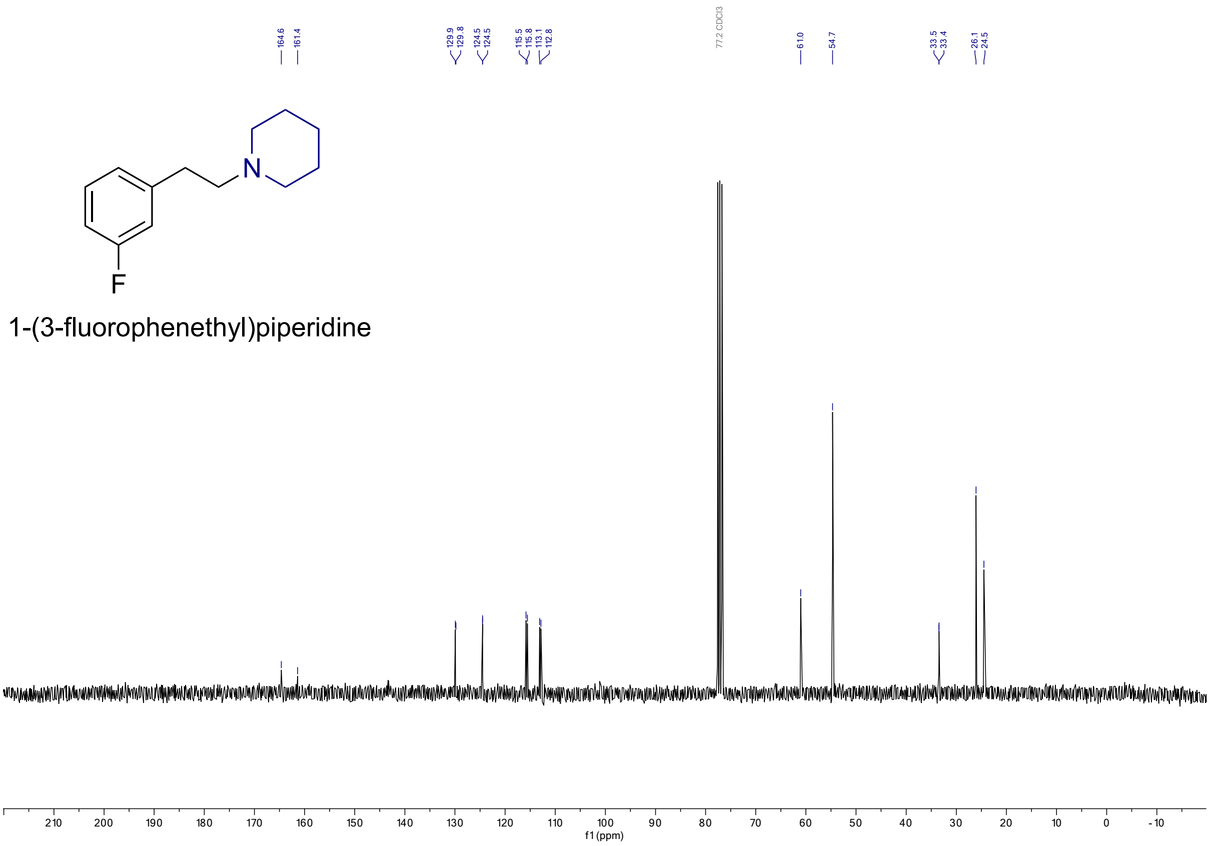

Figure S 30. $^{13}C\{^{1}H\}$ *NMR spectrum of* ***1e*** *in* $CDCl_3$ *(Spectrometer: 300 MHz).*

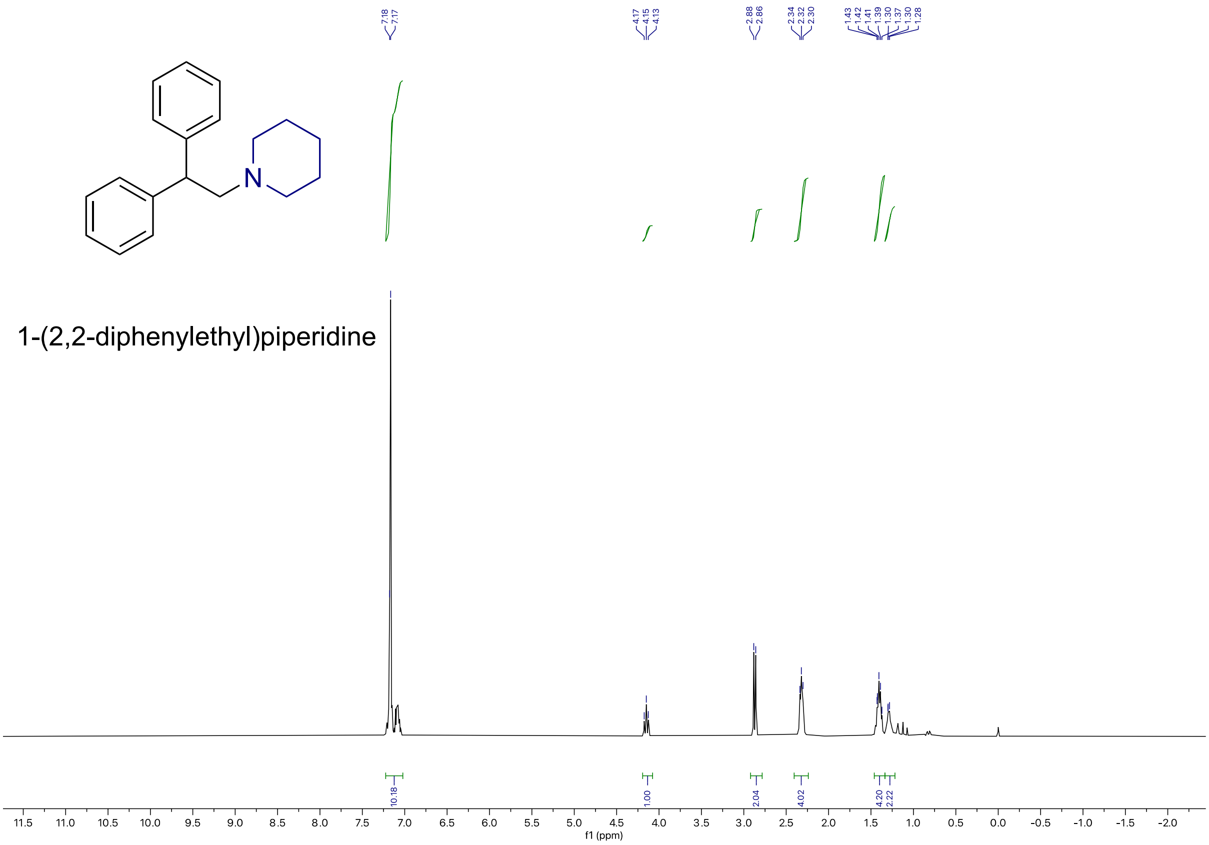

Figure S 31. ^{1}H NMR spectrum of ***1f*** *in $CDCl_3$ (Spectrometer: 300 MHz).*

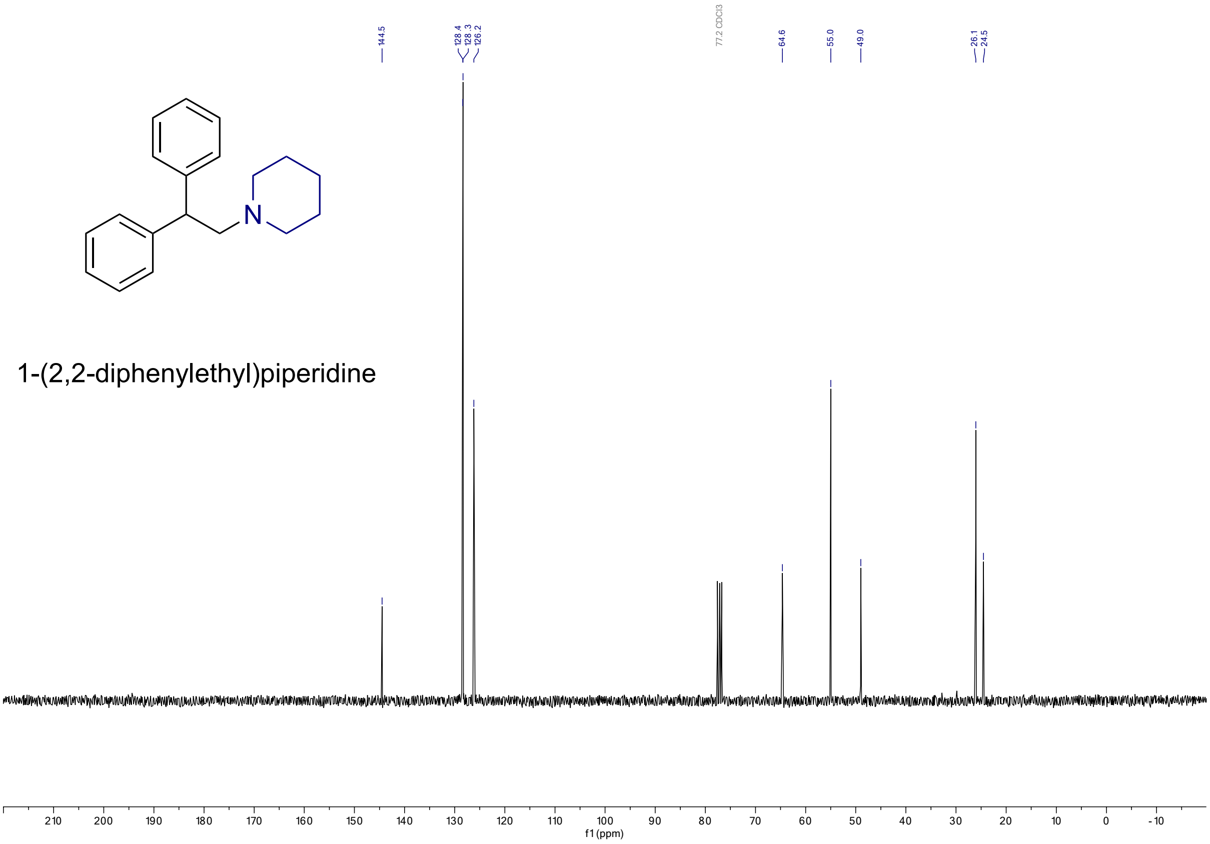

Figure S 32. $^{13}C\{^{1}H\}$ NMR spectrum of ***1f*** *in $CDCl_3$ (Spectrometer: 300 MHz).*

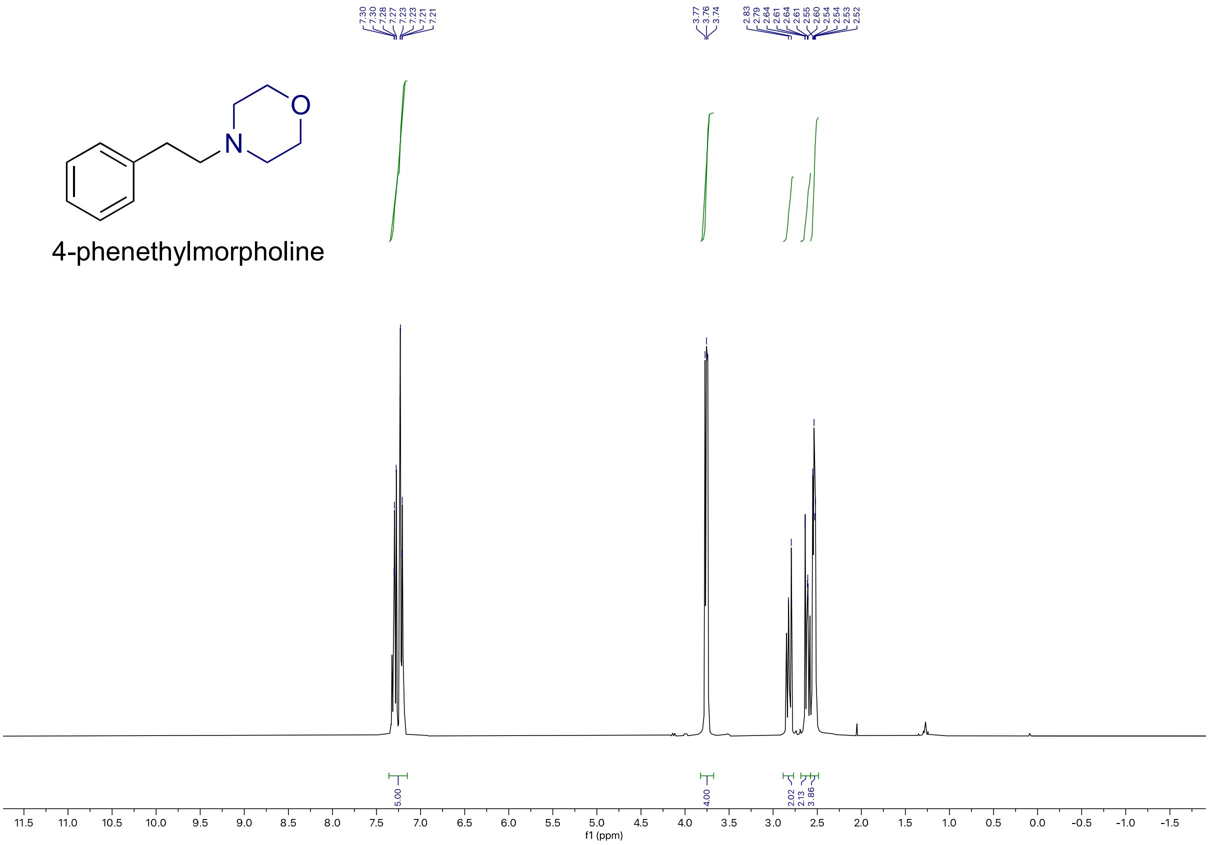

Figure S 33. [1]*H NMR spectrum of* ***1g*** *in* $CDCl_3$ *(Spectrometer: 300 MHz).*

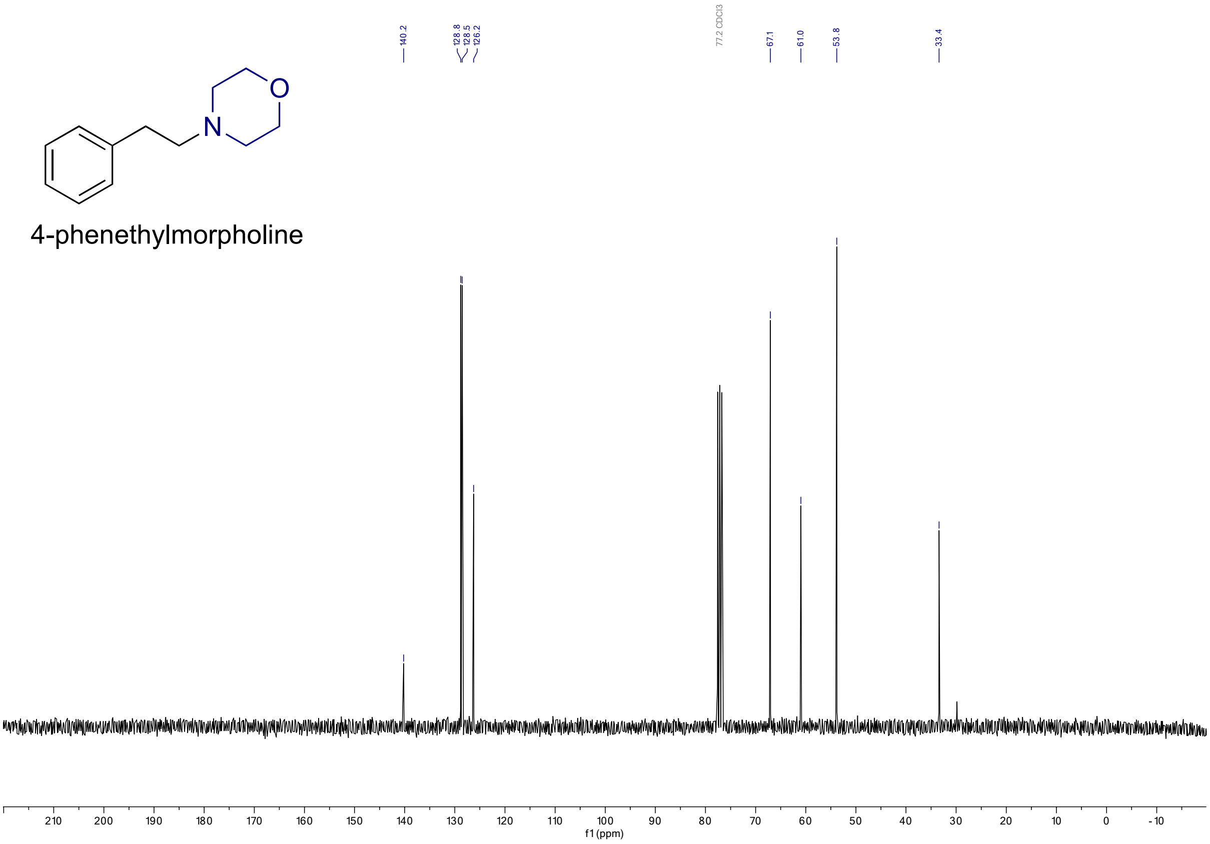

Figure S 34. [13]*C{*[1]*H} NMR spectrum of* ***1g*** *in* $CDCl_3$ *(Spectrometer: 300 MHz).*

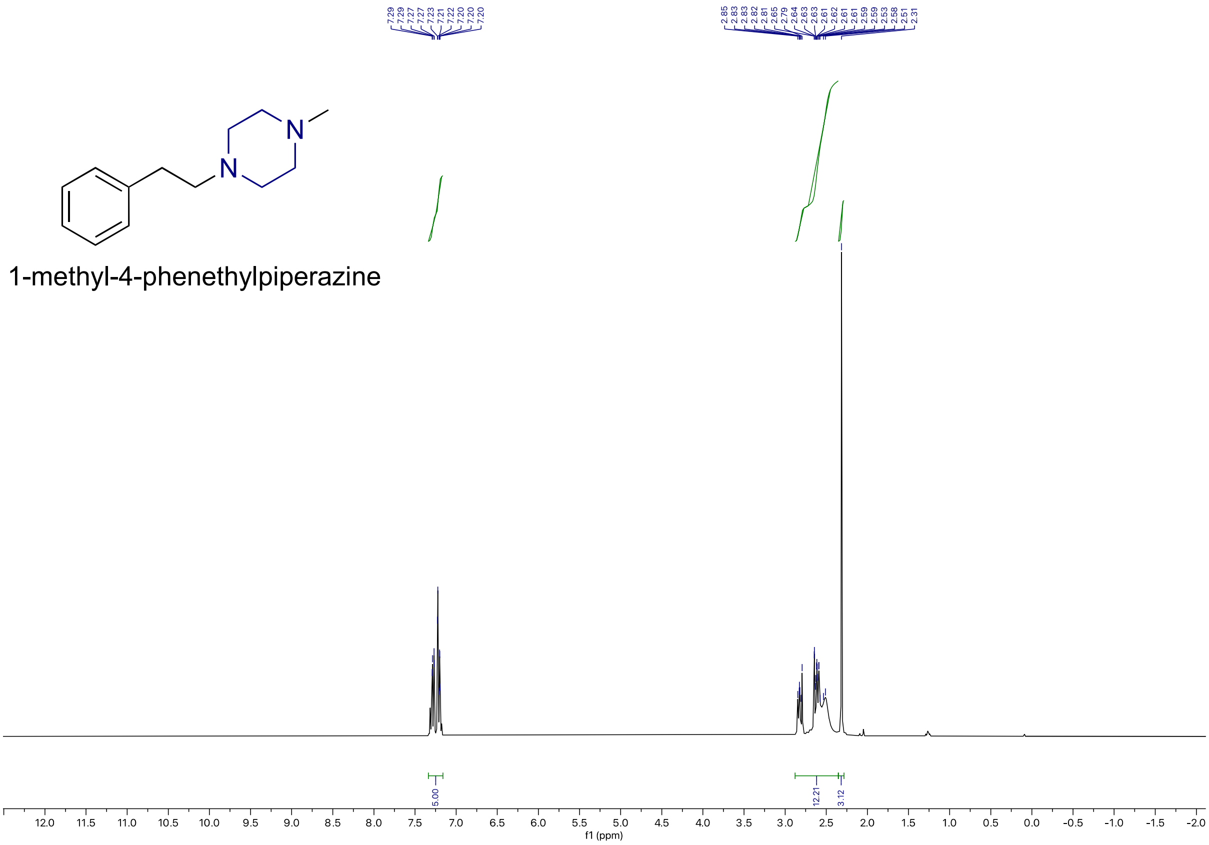

Figure S 35. ^{1}H *NMR spectrum of* ***1h*** *in* $CDCl_3$ *(Spectrometer: 300 MHz).*

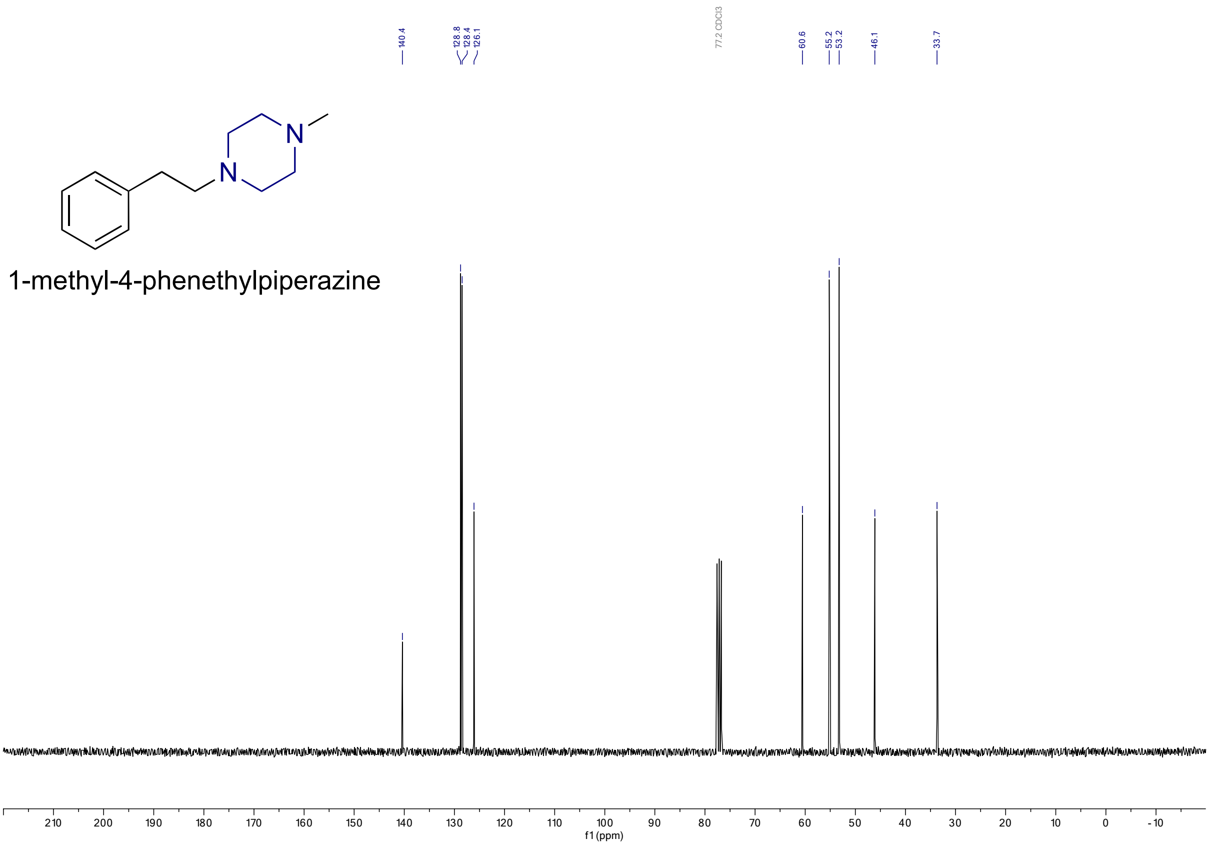

Figure S 36. $^{13}C\{^{1}H\}$ *NMR spectrum of* ***1h*** *in* $CDCl_3$ *(Spectrometer: 300 MHz).*

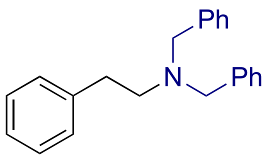

N,*N*-dibenzyl-2-phenylethan-1-amine

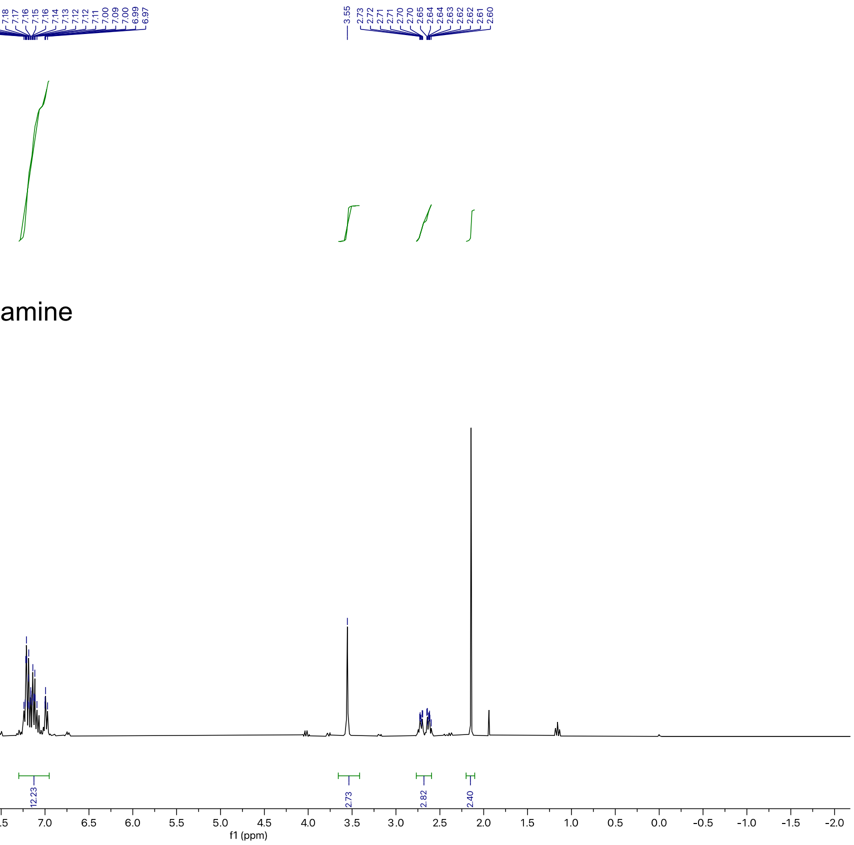

Figure S 37. ^{1}*H NMR spectrum of* ***1i*** *in* $CDCl_3$ *.* C_6Me_6 *(signal at 2.24 ppm) added as internal standard for NMR yield calculation.(Spectrometer: 300 MHz)*.

References

1. Tortajada, A.; Anderson, D. E.; Hevia, E. *Helvetica Chimica Acta* **2022**, *105* (8), e202200060. https://doi.org/10.1002/hlca.202200060.
2. Alborés, P.; Carrella, L. M.; Clegg, W.; García-Álvarez, P.; Kennedy, A. R.; Klett, J.; Mulvey, R. E.; Rentschler, E.; Russo, L. *Angew. Chem. Int. Ed.* **2009**, *48* (18), 3317-3321. https://doi.org/10.1002/anie.200805566.
3. Kottke, T.; Stalke,D. *J. Appl. Cryst.* **1993**, *26*, 615–619;
4. Stalke,D. *Chem. Soc. Rev.* **1998**, *27*, 171–178.
5. Oxford Diffraction **2018**. *CrysAlisPro* (Version 1.171.40.37a). Oxford Diffraction Ltd., Yarnton, Oxfordshire, UK.
6. Sheldrick, G. M. *Acta Cryst*. **2015** *A71*, 3-8.
7. Sheldrick, G. M. *Acta Cryst*. **2015** *C71*, 3-8.
8. Dolomanov, O.V.; Bourhis, L.J.; Gildea, R.J; Howard, J.A.K.; Puschmann, H. *J. Appl. Cryst.* **2009** *42*, 339-341.
9. Mulks, F. F.; Bole, L. J.; Davin, L.; Hernán-Gómez, A.; Kennedy, A.; García-Álvarez, J.; Hevia, E. *Angew. Chem. Int. Ed.* **2020**, *59* (43), 19021–19026. https://doi.org/10.1002/anie.202008512.
10. Zhang, X.-Y.; Zhai, D.-D.; Liu, Y.-F.; Guan, B.-T. *J. Organomet. Chem.* **2022**, *961*, 122254. https://doi.org/10.1016/j.jorganchem.2022.122254.
